# PARP inhibitors trap PARP2 and alter the mode of recruitment of PARP2 at DNA damage sites

**DOI:** 10.1093/nar/gkac188

**Published:** 2022-03-29

**Authors:** Xiaohui Lin, Wenxia Jiang, Johannes Rudolph, Brian J Lee, Karolin Luger, Shan Zha

**Affiliations:** Institute for Cancer Genetics, Vagelos College for Physicians and Surgeons, Columbia University, New York City, NY10032, USA; Institute for Cancer Genetics, Vagelos College for Physicians and Surgeons, Columbia University, New York City, NY10032, USA; Department of Biochemistry, University of Colorado Boulder, Boulder, CO80309, USA; Institute for Cancer Genetics, Vagelos College for Physicians and Surgeons, Columbia University, New York City, NY10032, USA; Department of Biochemistry, University of Colorado Boulder, Boulder, CO80309, USA; Howard Hughes Medical Institute, University of Colorado Boulder, Boulder, CO80309, USA; Institute for Cancer Genetics, Vagelos College for Physicians and Surgeons, Columbia University, New York City, NY10032, USA; Department of Pathology and Cell Biology, Herbert Irvine Comprehensive Cancer Center, Vagelos College for Physicians and Surgeons, Columbia University, New York City, NY10032, USA; Division of Pediatric Hematology, Oncology and Stem Cell Transplantation, Department of Pediatrics, Vagelos College for Physicians and Surgeons, Columbia University, New York City, NY10032, USA; Department of Immunology and Microbiology, Vagelos College for Physicians and Surgeons, Columbia University, New York City, NY10032, USA

## Abstract

Dual-inhibitors of PARP1 and PARP2 are promising anti-cancer drugs. In addition to blocking PARP1&2 enzymatic activity, PARP inhibitors also extend the lifetime of DNA damage-induced PARP1&2 foci, termed trapping. Trapping is important for the therapeutic effects of PARP inhibitors. Using live-cell imaging, we found that PARP inhibitors cause persistent PARP2 foci by switching the mode of PARP2 recruitment from a predominantly PARP1- and PAR-dependent rapid exchange to a WGR domain-mediated stalling of PARP2 on DNA. Specifically, PARP1-deletion markedly reduces but does not abolish PARP2 foci. The residual PARP2 foci in PARP1-deficient cells are DNA-dependent and abrogated by the R140A mutation in the WGR domain. Yet, PARP2-R140A forms normal foci in PARP1-proficient cells. In PARP1-deficient cells, PARP inhibitors - niraparib, talazoparib, and, to a lesser extent, olaparib - enhance PARP2 foci by preventing PARP2 exchange. This trapping of PARP2 is independent of auto-PARylation and is abolished by the R140A mutation in the WGR domain and the H415A mutation in the catalytic domain. Taken together, we found that PARP inhibitors trap PARP2 by physically stalling PARP2 on DNA via the WGR-DNA interaction while suppressing the PARP1- and PAR-dependent rapid exchange of PARP2.

## INTRODUCTION

PARP1 and PARP2 are DNA damage activated poly-ADP-ribose polymerases (PARPs), which catalyze the transfer of the ADP-ribose unit from the nicotinamide adenine dinucleotide (NAD^+^) to target proteins, including themselves and histones (1). The Poly-ADP-Ribose (PAR) chain (2) is highly charged, promotes chromatin relaxation, and directly recruits other DNA repair proteins, including, but not limited to, XRCC1 and its partner DNA Ligase 3 (LIG3) (3–6). Clinical PARP inhibitors inhibit the enzymatic activity of both PARP1 and PARP2, prevent DNA damage-induced PAR formation, delay DNA repair, and cause the accumulation of DNA single-strand breaks (SSB). In proliferating cells, these SSBs can be converted to DNA double-strand breaks (DSBs) during replication. The homologous recombination (HR) pathway plays an important role in replication-associated DSBs repair. Therefore, HR-deficient cancer cells (*e.g*., BRCA1 or BRCA2 mutated) are hypersensitive to PARP inhibition (7,8). Four PARP inhibitors have been approved for the treatment of BRCA1- or BRCA2-deficient cancers (9), leading to promising clinical responses. Among PARP1 and PARP2, PARP1 is responsible for ∼80% of DNA damage induced PARylation in mammalian cells. While the loss of either Parp1 or Parp2 alone is compatible with murine embryonic development, loss of both leads to embryonic lethality, suggesting critical overlapping functions. Moreover, loss of Parp2 in mouse models causes defects in T cell development (10,11), erythropoiesis (12,13), and spermatogenesis (14) that are not evident in *Parp1* null mice, suggesting unique functions of PARP2. In this context, a recent study suggests that PARP2 preferentially builds branched PAR chains, rather than straight PAR chains, which might attract different PAR-binding proteins. For example, Aprataxin and PNK-like factor (APLF), a chromatin bound DNA damage response factor with nuclease activity, preferentially binds to branched PAR chains (15).

Upon DNA damage, PARP1 and PARP2 are recruited to the DNA damage sites and surrounding chromatin. PARP1 and PARP2 foci form rapidly and transiently, with the intensity peaking within 1 min after damage and decreasing within 10 min (16,17). In addition to blocking the enzymatic activity of PARP1 and PARP2, PARP inhibitors also extend the appearance of chromatin bound PARP1 and PARP2 (17), and the appearance of DNA damage-induced PARP1 and PARP2 at UV-laser stripes (18) or micro-irradiation foci (16), collectively termed trapping. Trapping is critical for the anti-cancer effects of PARP inhibitors (19,20). As such, the cytotoxicity of PARP inhibitors correlates with their ability to trap PARP1 and 2, but not with their IC50 for enzymatic inhibition (21). Both PARP1 and PARP2 have a modular organization, with DNA binding domains at the N-terminus, followed by a conserved Tyr-Gly-Arg (WGR) domain, and a C-terminal catalytic domain (CAT). Structural analyses show that DNA binding by the three zinc-finger (ZF) domains of PARP1 triggers allosteric changes through the WGR domain, leading to PARP1 activation (22,23). While both the unstructured N-terminal region (NTR) and the WGR domain of PARP2 can bind to DNA, only the WGR domain is essential for DNA-induced activation of PARP2 (24). PARP1 and PARP2 share a high degree of homology within the CAT domain, including the conserved H-Y-E catalytic triad that interacts with NAD^+^ and PARP inhibitors, and is essential for the enzymatic activity of PARP1 and PARP2 (25–27). *In vitro*, NAD^+^ triggers auto-PARylation and the release of both PARP1 and PARP2 from DNA (24,28). To understand the nature of PARP1 trapping *in vivo*, we previously developed the live-cell imaging and fluorescence recovery after photobleaching (FRAP) assays to characterize the dynamics of PARP1 upon 405 nm micro-irradiation. Our results showed that PARP1 exchanges rapidly at the site of DNA damage with or without PARP inhibitors (16). We proposed that the persistent PARP1 foci were formed by the continuous recruitment of different PARP1 molecules to the DNA lesions due to delayed repair (16). Accordingly, loss of XRCC1 also causes persistent PARP1 foci without affecting PARP1 exchange. Moreover, unlike the non-hydrolyzable NAD^+^ analog benzamide adenine dinucleotide (BAD), clinical PARP inhibitors cannot allosterically lock purified PARP1 on model DNA substrates (29).

In addition to PARP1, clinical PARP inhibitors also extend the appearance of DNA damage-induced PARP2 stripes (18) or chromatin bound PARP2 (17). To understand the mechanism of PARP2 trapping, here we characterized the impact of PARP inhibitors on PARP2 foci using live-cell imaging. Our results identified two mechanisms for PARP2 foci formation – one that is PARP1-dependent and requires the PAR chain but not the WGR domain, and another that is PARP1-independent and requires the WGR domain. In the absence of PARP inhibitors, the PARP1-dependent mechanism contributes to the majority of PARP2 foci formation. PARP inhibitors prevent PAR formation and reduce the PARP1 dependent recruitment of PARP2, while physically trapping PARP2 at the DNA damage site to enhance the PARP1-independent recruitment of PARP2. Mechanistically, the inhibitor-mediated trapping of PARP2 is independent of its own PARylation activity but requires the R140 at the WGR domain and H415 in the CAT domain. Taken together, our findings indicate that PARP inhibitors cause delayed yet persistent PARP2 foci by switching the mode of PARP2 recruitment from predominantly PAR and PARP1 dependent rapid exchange to DNA and WGR domain-dependent stalling.

## MATERIALS AND METHODS

### Mouse alleles and Parp1 conditional mice

The *Parp2* conditional (30) and Rosa26a-ERCreT2 alleles were previously described (31). Loss of both *Parp1* and *Parp2* lead to embryonic lethality in mice (32). To generate *Parp1* and *Parp2* double deficient cells and study the impact of dual *Parp1 and 2* loss in somatic tissues, we generated a *Parp1* conditional allele (Supplementary Figure S1A) by placing two loxP sites flanking exon 4 of murine *Parp1* in a 129Sv murine embryonic stem cell line (CSL5). The successful targeting was confirmed by Southern blotting with a 3′ probe (Supplementary Figure S1A). Upon ScaI digestion, the germline allele migrated at 8.2 kb and the targeted allele migrated at 5.5 kb (Supplementary Figure S1A). Two independently targeted clones were injected for germline transmission and the resulting chimera were bred with the constitutive FLIPase expressing mice to remove the neo-resistant cassette. Recombination between the two LoxP sites removes exon 4 and causes a frameshift and early termination in the ZnF domain of the murine Parp1. The absence of the Parp1 protein was verified by western blotting (Supplementary Figure S1B). The following primers were used to genotype the *Parp1* conditional allele and the corresponding *Parp1* deleted allele (5′-TGCTAGGGACCAGCAGAACT-3′, 5′-CCATGCTCATCAGCGACACC-3′ and 5′-GGCCTGCTTCTACTACCTCC-3′). PCR was carried out under the following conditions: 94ºC for 5 min followed by 32 cycles 94ºC 30 s, 62.5ºC 30 s, 72ºC 30 s. The product corresponding to the wild-type allele is ∼280 bp, the conditional allele is ∼370 bp, and the deleted allele is ∼600 bp.

### Cell lines and cell culture

The TERT-immortalized human retinal pigment epithelial-1 (RPE-1) cells of wild type (WT), *PARP1* knockout (KO), *PARP2* KO, and *PARP1/2* double knockout (DKO) were generously provided by Dr Keith W. Caldecott at the University of Sussex (33) and cultured in DMEM medium (GIBCO, Cat. 12430062) supplemented with 10% fetal bovine serum (FBS), MEM non-essential amino acids (GIBCO, Cat. 11140050), 2 mM glutamine, 1 mM sodium pyruvate and 50 U/ml penicillin/streptomycin (GIBCO, 15140122). The WT, *Parp1* KO, *Parp2* KO, and *Parp1/2* DKO immortalized murine embryonic fibroblast (iMEFs) were isolated (as single clones) from 4-hydroxytamoxifen (4OHT, 200 nM, 48 h) treated *Rosa26a^Cre-ERT2/+^Parp1^C/C^*, *Rosa26^Cre-ERT2/+^Parp2^C/C^* and *Rosa26^Cre-ERT2/+^Parp1^C/C^Parp2^C/C^* iMEFs. The successful deletion was verified by PCR and western blotting for Parp1 and Parp2 proteins (Supplementary Figure S1B). Primary MEFs were derived from a timed breeding using a standard protocol. The cells were then immortalized via SV40 large and small antigens as previously described (34).

### Chemicals and antibodies

The PARP inhibitors olaparib (Selleckchem, S1060), niraparib (Selleckchem, S2741), and talazoparib (Selleckchem, S7048) were dissolved in dimethyl sulfoxide (DMSO) and used at 1 μM final concentration. Anti-PARP1 antibody (CST, 9542) was used at 1:5000. Anti-PARP2 antibody (Active Motif, 39044) was used at 1:2000. Anti-XRCC1 antibody (Novus Biologicals, 87154) was used at 1:5000. Anti-PAR antibody (R&D, 4335-MC-100) was used at 1:1000. Anti-Histone H3 antibody (Abcam, ab1791) was used at 1:5000. Anti-α-Tubulin antibody (Sigma, CP06) was used at 1:5000. Anti-β-Actin antibody (Sigma, A5441) was used at 1:10 000. 8-MOP (Methoxsalen) (Sigma, M3501) was added 10 min before micro-irradiation at a final concentration of 100 μM when used.

### Plasmids

The DsRed-mono-C1-XRCC1 and pEGFP-C1-PARP2 plasmids were generously provided by Dr Li Lan at Massachusetts General Hospital (35) and Dr Xiaochun Yu at Westlake University (36), respectively. The pEGFP-C1-PARP2-H415A, -E545A and -R140A plasmids were generated by direct mutagenesis of the pEGFP-C1-PARP2. pEGFP-C1-SV40NLS-ΔNTR-PARP2 (1–70aa deletion) was generously provided by Dr John M. Pascal at the University of Montreal (24). pEGFP-C1-SV40NLS-PARP2^88-570^ (large) was generated by replacing the coding sequence of ΔNTR-PARP2 with the coding sequence of 88–570aa of human PARP2. All mutations were validated via Sanger sequencing.

### Chromatin fractionation assay

RPE-1 cells were treated with or without 1 μM niraparib for 1 h in the presence of 0.1 mg/ml MMS. Cells were then harvested and lysed in lysis buffer containing 100 mM KCl, 2.5 mM MgCl2, 5 mM EDTA, 50 mM HEPES, 3 mM dithiothreitol, 10% glycerol, 0.5% Triton X-100, and protease inhibitor cocktail (Roche, 56079100) for 45 min on ice with tapping every 15 min. Lysates were centrifuged at ∼16 000 g for 15 min at 4°C. Supernatants containing soluble proteins were collected and the pellet containing chromatin bound proteins was washed twice with lysis buffer, then subjected to sonication for 10 min. The chromatin and soluble extracts were mix with SDS-PAGE loading buffer and incubated at 95°C for 7 min. Then samples were subjected to Western Blotting with indicated antibodies.

### PARP inhibitor sensitivity assay

The RPE-1 cells were seeded at 400 cells per well into 96-well plates and treated with niraparib at different concentrations at 3 h after initial seeding. The cell proliferation activity was measured at day 6 after mock or niraparib treatment using the Cell Proliferation Kit I (MTT) (Roche, 11465007001). The absorbance of formazan product and the reference were measured at 550 and 650 nm, respectively on a GloMax^®^-Multi + Microplate Multimode Reader (Promega, WI, USA) and plotted as a dose-response curve using GraphPad Prism. To express Empty-IRES-hCD8 and PARP2^RA^-IRES-hCD8 in the *PARP1/2* DKO RPE-1 cells, the cassettes were packed into retrovirus and positively infected cells were purified with anti-hCD8 MACS beads as describe before (37).

### Live-cell Imaging data collection and processing

Live-cell imaging analyses were performed as described before (16) with minor modifications. Briefly, ∼5 × 10^4^ RPE-1 cells or MEFs were seeded onto each 35 mm diameter glass-bottom plates on day 1. On day 2, the cells were transfected with plasmids encoding fluorescence protein-tagged PARP2 and/or XRCC1 via Lipofectamine 2000 (Invitrogen, Cat. 11668019) or Lonza 4D-Nucleofector™ X according to manufacturer instructions. Live-cell imaging was performed 24 h after transfection with a Nikon Ti Eclipse inverted microscope (Nikon Inc, Tokyo, Japan) equipped with the A1 RMP confocal microscope system and Lu-N3 Laser Units (all from Nikon Inc.). Only cells with moderate yet reliable expression (∼200–1000 a.u.) of the GFP- or RFP- tagged protein were imaged. Laser micro-irradiation and timelapse imaging were carried out using the NIS Element High Content Analysis software (Nikon Inc.) and a 405 nm laser (energy level ∼500 uW for a ∼0.8 μm diameter region).

For the short-term kinetics assay, images were acquired every 10 s after micro-irradiation for a total of 5 min. For the long-term kinetics assay, images were acquired at 1, 5, 10, 20 and 30 min after micro-irradiation. The relative intensity at damaged sites was calculated as the ratio of the mean intensity at each micro-irradiation damaged site to the corresponding mean intensity of the nucleus as background. For FRAP, GFP-PARP2 foci were photobleached with a 488 nm laser specific for GFP, and the fluorescence recovery was captured every 5 s for a total of 2 min. Normalized fluorescence intensity for each time point was determined by setting the intensity immediately before and after photobleaching as 100% and 0%, respectively. Fiji software was used for quantifications and at least eight individual cells were analyzed for each data point in each experiment. At least two independent experiments were conducted. The maximal recovery is defined as the ratio between the recovered intensity at the infinite time (plateau) after bleaching and the fluorescence intensity in the bleached area before bleaching, while *t*_1/2_ is defined as the time needed for the fluorescence level to reach 50% of the maximum recovered intensity (38).

### Expression, purification and characterization of PARP2

hPARP2 and mPARP2 were expressed and purified as previously described (28). Circular dichroism to validate proper folding was performed for all constructs of PARP2 (hPARP2 and H414A and E534A mutants of mPARP2) on a Chirascan Plus (Applied Photophysics) CD spectrometer at 0.2 mg/ml protein in 20 mM Potassium Phosphate (pH 7.5), 0.1 mM EDTA, 0.1 mM TCEP. All spectra are reported in Mean Residue Ellipticity. Recombinant HPF1 was purified as previously described (39).

### Fluorescence polarization assays to measure DNA binding to PARP2

PARP2 was serially diluted in binding buffer (50 mM Tris–HCl (pH 8.0), 150 mM NaCl, and 0.01% IGEPAL) from 4 μM to 90 nM in a volume of 10 μl of a 384-well plate (Corning 3575). Fluorescently labeled pNick (10 μl, 5 nM, 5′-phosphate-GCT GAG CTT CTG GTG AAG CTC AGC TCG CGG CAG CTG GTG CTG CCG CGA-3′) was added to each well and incubated for 30 min to ensure complete association. All concentrations cited are final concentrations in a volume of 20 μl. Fluorescence polarization (FP) using excitation at 482 nm (bandwidth 16 nm), dichroic filter at 496 nm, and emission at 530 nm (bandwidth 40 nm) was monitored from the top of the plate using a BMG Labtech CLARIOstar plate reader.

### Fluorescence polarization assays to measure the activity of PARP2

PARP2 (10 nM, WT versus mutants) was diluted in binding buffer in the presence of fluorescein-labeled pNick (7.5 nM) across seven wells of a 384-well plate (Corning 3575) in a volume of 20 ul and pre-incubated for 30 min to ensure complete association. Dissociation of labeled DNA was initiated by the addition of 10 μl of varying concentrations of NAD^+^ (63 μM–2 mM). All concentrations cited are final concentrations in a volume of 30 μl. Control wells lacked NAD^+^. Fluorescence polarization (FP) was monitored as in the DNA binding assays, but in a time-dependent mode with 7 s intervals over the course of 45 min. Assays in the presence of HPF1 were performed using the injector mode and 1 s read intervals on the CLARIOstar plate reader in the presence of 200 μM NAD+ and 2 μM HPF1 over the time course of 4 min.

## RESULTS

### PARP1 plays an important role in the formation of DNA damage-induced PARP2 foci

**Figure 1.**
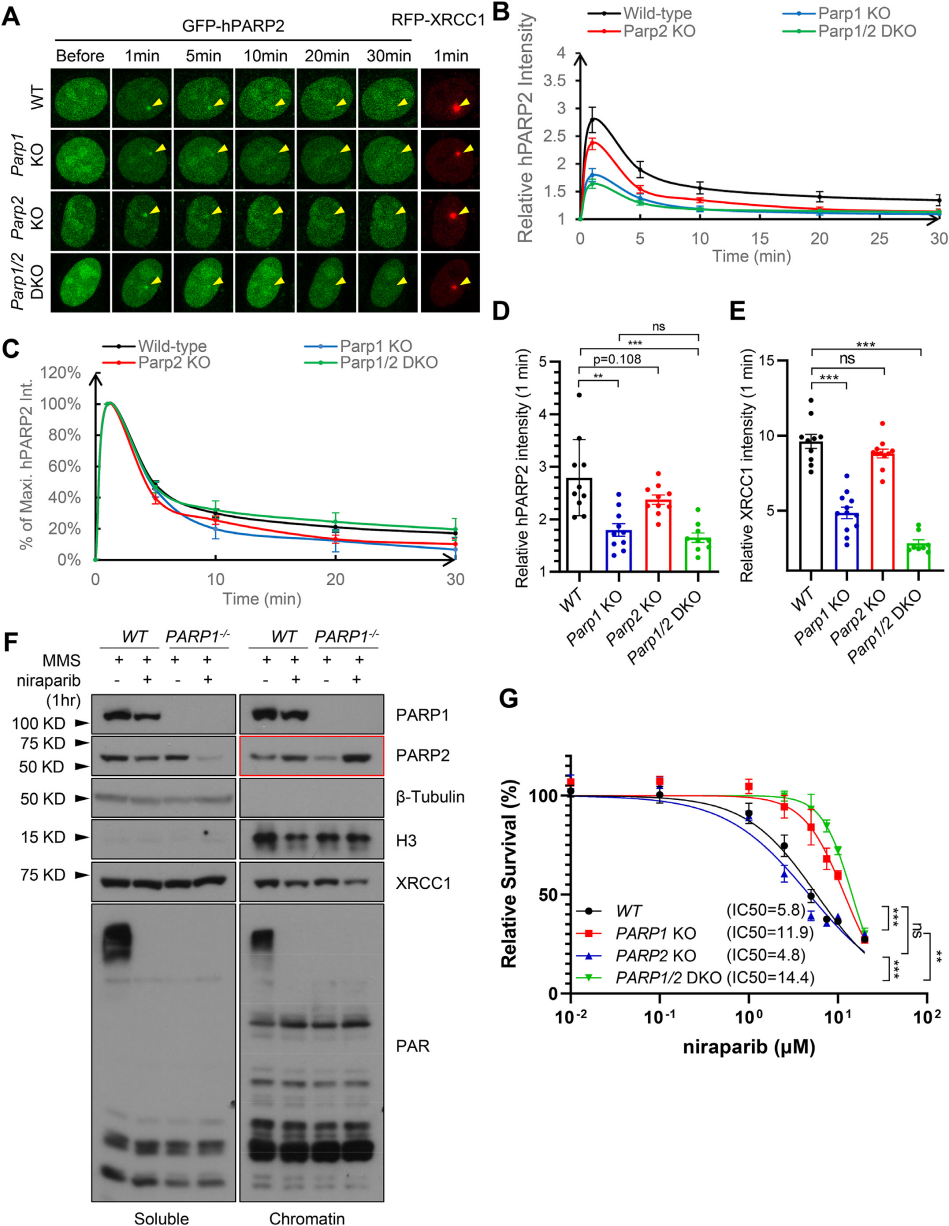
PARP1 promotes PARP2 recruitment at DNA damage sites and niraparib enhances PARP2 foci independent of PARP1. (**A**) Representative images of laser-induced GFP-PARP2 and mRFP-XRCC1 foci in WT, *Parp1* KO, *Parp2* KO, and *Parp1/2* DKO iMEF cells. The yellow arrowheads point to the area of micro-irradiation. (**B**) The relative intensity kinetics and (**C**) The normalized kinetics (plotted as the percentage of the maximal relative intensity at 1 min) of GFP-PARP2 at DNA damage sites in WT, *Parp1* KO, *Parp2* KO and *Parp1/2* DKO iMEF cells. (**D**, **E**) The relative intensity of (B) GFP-PARP2 and (C) RFP-XRCC1 at 1 min post-microirradiation. Two-tailed unpaired Student's *t*-test were used to calculate the p-values. (**F**) Soluble and chromatin fraction of WT or *PARP1* KO RPE-1 cells treated with MMS (0.1 mg/ml, 1 h) and niraparib (1 μM, 1 h). (**G**) The niraparib sensitivity of WT, *PARP1* KO, *PARP2* KO, and *PARP1/2* DKO RPE-1 cells. The dots and error bars represent means and SEM, respectively, from one representative experiment out of three consistent biological repeats with triplicate samples (for sensitivity) and at least 8–10 cells (for live cell imaging) per experiment. *P* value of IC50 was calculated using the extra sum-of-square *F* test. ns, *P* > 0.05; **P < 0.01; ***P < 0.001.

To study the kinetics of PARP2 at DNA damage sites, we established a live-cell imaging system in which we monitored GFP-tagged PARP2 foci upon micro-irradiation with a 405 nm laser without sensitization (16). We first assessed the impact of endogenous PARP1 and PARP2 on the formation of PARP2 foci by comparing GFP-PARP2 foci in immortalized wild-type (WT), *Parp1* KO, *Parp2* KO and *Parp1/2* DKO iMEFs. XRCC1 is recruited to the DNA damage foci by directly binding to PAR (40), so mRFP-tagged-XRCC1 was used as an indicator of the PAR levels. The intensity of PARP2 foci peaked at the first time point measured—1 min after micro-irradiation—and decreased rapidly by 5 min (to ∼40% of peak intensity) regardless of the presence or absence of endogenous Parp1 or Parp2 (Figure 1A–C). While endogenous Parp2 (in WT versus *Parp2* KO) does not statistically significantly affect GFP-Parp2 foci intensity, loss of endogenous Parp1 (in *Parp1* KO and *Parp1/2* DKO cells) decreases Parp2 foci intensity by ∼2/3 (Figure 1D). Accordingly, MMS-induced chromatin bound PARP2 is also more prominent in *PARP1 WT* hTERT-immortalized human retinal pigment epithelial (RPE-1) cells than in *PARP1* KO isogenic control cells (Figure 1F). Notably, *PARP1* KO reduces the maximal intensity of PARP2 foci without altering the decay kinetics of PARP2 foci (Figure 1C). PARP1 is responsible for 80–90% of DNA damage-induced PARylation (41). Correspondingly, XRCC1 foci intensity reduced significantly with PARP1 loss, and further reduced upon the loss of both PARP1 and PARP2 (Figure 1E). The reduction of Parp2 foci intensity in *Parp1* KO cells is similar in magnitude to the reduction of XRCC1 foci intensity in the same cell (Figure 1D, and E), consistent with the notion that PARP1 recruits PARP2 via a PAR dependent mechanism as proposed before (15).

### PARP inhibitors delay but do not abolish PARP2 foci formation in PARP1 proficient cells

To formally test whether PARP1 protein or its PARylation activity promotes PARP2 foci formation, we treated the cells with niraparib, a clinically used dual PARP1/2 inhibitor (42). Consistent with trapping, niraparib increased chromatin bound PARP2 in WT cells (Figure 1F). Moreover, we noted that niraparib treated *PARP1* KO cells have the highest levels of chromatin bound PARP2, and as such, the entire soluble fraction is almost depleted of PARP2 (Figure 1F). This is unexpected, given we found that PARP1 is responsible for the majority of PARP2 foci. In this context, loss of PARP1, but not loss of PARP2 alone, confers resistance to niraparib (Figure 1G). Additional loss of PARP2 in *PARP1* KO cells caused a notable, yet mild resistance to niraparib, suggesting niraparib might be able to trap PARP2 independent of PARP1.

**Figure 2.**
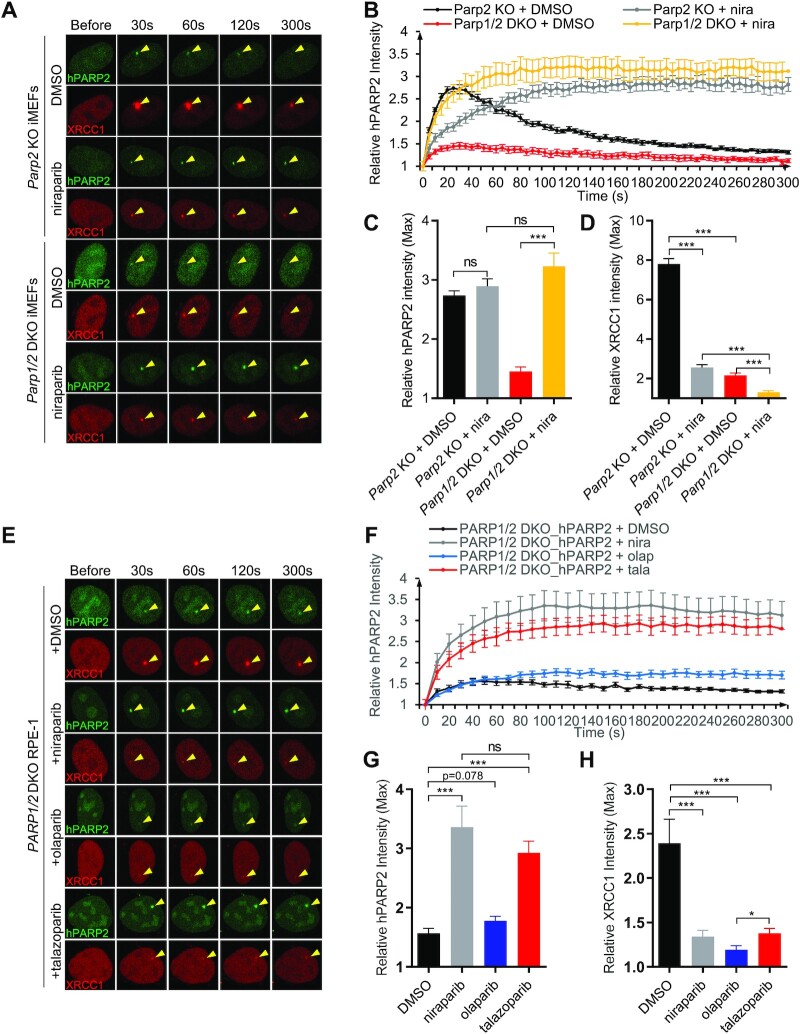
Niraparib enhances PARP2 foci in PARP1-deficient cells. (**A**) Representative live-cell images of GFP-PARP2 and mRFP-XRCC1 and (**B**) The relative intensity kinetics of PARP2 at DNA damage sites in *PARP2* KO and *PARP1/2* DKO cells in the presence and absence of niraparib. (C and D) The maximal relative intensity of (**C**) GFP-PARP2 and (**D**) mRFP-XRCC1. (**E**) Representative images of GFP-PARP2 and mRFP-XRCC1 and (**F**) the relative intensity kinetics of PARP2 at DNA damage sites in *PARP1/2* DKO RPE-1 cells in the presence and absence of PARP inhibitors (niraparib, olaparib and talazoparib). (**G**, **H**) The maximal relative intensity of (G) GFP-PARP2 and (H) mRFP-XRCC1. The dots and bars represent means and SEM, respectively, from one representative experiment out of consistent 2–4 biological repeats with *n* > 8 cells per experiment. Two-tailed unpaired Student's *t*-test was used. ns, *P* > 0.05; *P < 0.05; **P < 0.01; ***P < 0.001.

To avoid the confounding effects of endogenous Parp2, we performed the live-cell imaging experiments in *Parp2* KO MEFs. To better characterize the early kinetics of PARP2 foci, we collected images at 10 s (instead of 1 min) intervals for a total of 5 min (300 s) (Figure 2A and B). Under this condition, PARP2 foci intensity peaked at ∼30 s after micro-irradiation (Figure 2B). Consistent with the role of PAR in the damage-induced PARP2 foci formation (15), niraparib treatment delayed PARP2 foci formation in cells with endogenous PARP1 (peaks at ∼3 min with niraparib). However, after 3 min, PARP2 accumulated at high, if not higher, levels, in the presence of niraparib (Figure 2A–C). This delayed yet robust PARP2 foci formation in niraparib treated *Parp1^+/+^* cells cannot be explained by a role of PAR in PARP2 recruitment, since XRCC1 foci were extremely weak in niraparib-treated *Parp1*^*+/+*^ MEFs (Figure 2D and Supplementary Figure S1C). Similar to MEFs, PARP1-deletion, but not niraparib treatment, significantly reduced PARP2 foci in RPE-1cell line (Supplementary Figure S1E–H). Taken together, these results point out the existence of a PAR-independent mechanism for PARP2 foci formation.

### PARP inhibitors intensify the damage-induced PARP2 foci in Parp1-deficient cells

To determine whether the PAR-independent recruitment of PARP2 in niraparib-treated cells requires PARP1, we measured PARP2 foci formation in niraparib-treated *Parp1/2* DKO iMEF cells. Strikingly, niraparib increased PARP2 foci intensity >3-fold, reaching and exceeding the level in control *Parp1*-proficient cells (Figure 2A–C). The PARP2 foci intensity continued to increase for ∼100 s and stayed at a high level for the entire 5-min measurement window (Figure 2A, B, and Supplementary Figure S1D). The higher PARP2 foci intensity in niraparib-treated PARP1 deficient cells than in PARP1-proficient cells (Figure 2B, and C) suggests that endogenous PARP1 might compete with PARP2 for foci formation under this condition. To ensure this was not a cell line-specific effect, we repeated the experiment in a human epithelial cell line -RPE-1 cells. Indeed, PARP1 deletion also markedly enhanced GFP-PARP2 foci in *PARP1/2* DKO RPE-1 cells in the presence of niraparib (Supplementary Figure S1F–H). Correspondingly, niraparib also increased chromatin bound PARP2 in *PARP1* KO cells (Figure 1F). Moreover, this observation is not limited to niraparib, as another PARP inhibitor—talazoparib with strong trapping (18,29,43)—also markedly increased PARP2 foci in *PARP1/2* DKO RPE-1 cells (Figure 2E–G). Meanwhile, the extent of trapping does not correlate with enzymatic inhibition of PARP2. As such, olaparib, with a compariable IC50 for PARP2, shows relatively moderate trapping and did not significantly intensify the PARP2 foci in the *PARP1/2* DKO cells (Figure 2E–G). All three inhibitors effectively dimmed the XRCC1 foci (Figure 2E and H), consistent with the lack of PARylation.

### PARP inhibitors physically trap PARP2 at DNA damage sites in PARP1-deficient cells

**Figure 3.**
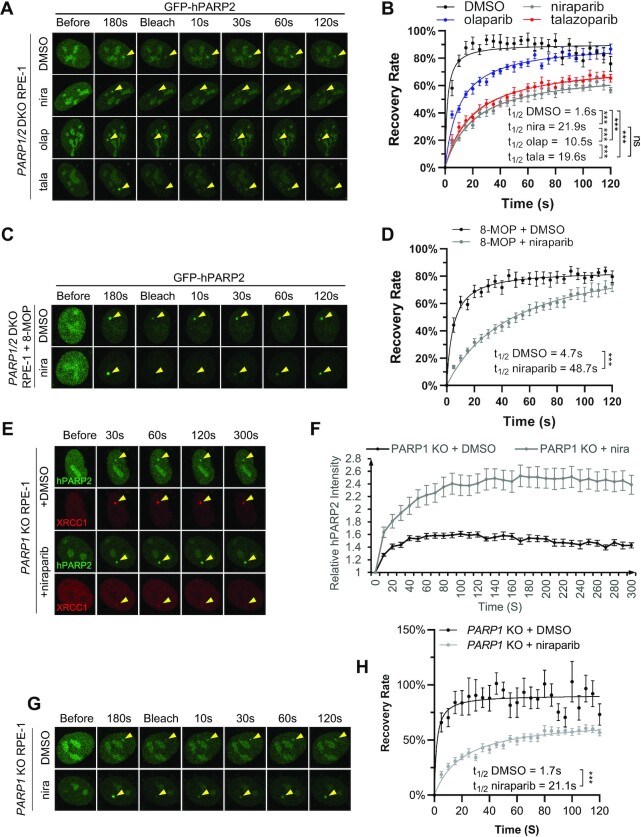
PARP inhibitors physically trap PARP2 at the DNA damage sites. (**A**) Representative images and (**B**) calculated FRAP recovery curves for GFP-PARP2 in *PARP1/2* DKO RPE-1 cells in the presence and absence of PARP inhibitors. *t*_1/2_ = 1.6 ± 0.7 s, *B*_max_= 90.3 ± 2.5% for DMSO; *t*_1/2_ = 21.9 ± 4.3 s, *B*_max_= 71.4 ± 4.0% for niraparib; *t*_1/2_ = 10.5 ± 1.8 s, *B*_max_= 90.9 ± 8.0% for olaparib; *t*_1/2_ = 19.6 ± 3.9 s, *B*_max_= 76.6 ± 4.0% for talazoparib. (**C**) Representative images and (**D**) calculated FRAP recovery curves for GFP-PARP2 in *PARP1/2* DKO RPE-1 cells treated with 8-MOP in the presence and absence of niraparib. *t*_1/2_ = 4.7 ± 1.2 s, *B*_max_= 84.1 ± 2.8% for DMSO; *t*_1/2_ = 48.7 ± 10.9 s, *B*_max_= 100.1 ± 9.5% for niraparib. (**E**) Representative images and (**F**) calculated kinetics for GFP-PARP2 in *PARP1* KO RPE-1 cells in the presence and absence of niraparib (1 μM). (**G**) Representative images and (**H**) calculated FRAP recovery curves for GFP-PARP2 in *PARP1* KO RPE-1 cells in the presence and absence of niraparib (1 μM). *t*_1/2_ = 1.7 ± 1.6s, *B*_max_= 90.8 ± 6.0% for DMSO; *t*_1/2_ = 21.1 ± 4.9s, *B*_max_= 69.33 ± 4.5% for niraparib. For (B), (D) and (H) *P* value was calculated using the extra sum-of-square *F* test. ns, *P* > 0.05; ***P < 0.001. All the dots and bars represent means and SEM, respectively, from one representative experiment out of 2–4 with *n* > 8 cells each time with consistent results.

To understand how niraparib causes enhanced PARP2 foci on damaged DNA, we performed FRAP. A 488 nm laser targeting GFP was used to photobleach the GFP-PARP2 foci. The recovery of the GFP-PARP2 after photobleaching reflects the recruitment of unbleached GFP-PARP2 that displaces the bleached PARP2 at the foci, and, by extension, rapid exchange of PARP2 (Figure 3A and B). When bleached at the time when the PARP2 foci intensity reached the plateau (∼180 s, Figure 2F), GFP-PARP2 foci recovered rapidly (*t*_1/2_ = 1.6 ± 0.7 s) and efficiently (maximal recovery = 90.3 ± 2.5%) in DMSO treated controls (Figure 3A and B). We note that the *t*_1/2_ of PARP2 seems significantly faster than that of PARP1 (*t*_1/2_ = 5.4 ± 1.6 s) (16) reported previously under the same experimental condition. Moreover, the recovery kinetics of PARP2 in DMSO-treated cells bleached at 60 s (peak intensity, *t*_1/2_ = 1.3 s) is very similar to those bleached at 180s after irradiation (Supplementary Figure S2B). In contrast, although PARP2 foci eventually recovered in the presence of niraparib (maximal recovery = 71.4 ± 4.0%), olaparib (maximal recovery = 90.9 ± 8.0%) or talazoparib (maximal recovery = 76.6 ± 4.0%), the kinetics of recovery were markedly delayed by niraparib (*t*_1/2_ = 21.9 ± 4.3 s, *P* < 0.0001 by extra sum-of-squares *F* test) and talazoparib (*t*_1/2_ = 19.6 ± 3.9 s, *P* < 0.0001) and relatively moderately delayed by olaparib (*t*_1/2_ = 10.5 ± 1.8 s, *P* < 0.0001) (Figure 3A and B). The extent of delay in PARP2 exchange correlates with their ability to intensify PARP2 foci in *PARP1/2* DKO cells (niraparib = talazoparib > olaparib, Figures 2F, 3B, and Supplementary Figure S2A). Treatment with photo-sensitizer 8-MOP increased the PARP2 and XRCC1 foci intensity (Supplementary Figure S2C) (44) but did not affect the ability of niraparib to trap PARP2 (*t*_1/2_ = 4.7 ± 1.2 s in control versus *t*_1/2_ = 48.7 ± 10.9 s with niraparib) (Figure 3C, D, and Supplementary Figure S2D), suggesting the different levels of trapping cannot be simply attributed to the different intensity of the initial PARP2 foci. Correspondingly, in 8-MOP pretreated *PARP1/2* DKO RPE-1 cells, niraparib treatment also enhanced PARP2, but not XRCC1 foci intensity (Supplementary Figure S2D–G). Moreover, we also measured the PARP2 foci kinetics and FRAP in *PARP1* KO cells with endogenous PARP2, and found that niraparib also enhanced GFP-PARP2 foci in *PARP1* KO cells (Figure 3E, F, and Supplementary Figure S2H) and delayed the exchange of PARP2 (Figure 3G, H and Supplementary Figure S2I). As expected, niraparib diminished XRCC1 foci in *PARP1* KO cells (Supplementary Figure S2J). Collectively, the data suggest that niraparib and talazoparib physically stall PARP2 at the DNA damage site, which explains the enhanced PARP2 foci upon treatment.

### The physical trapping of PARP2 does not correlate with enzymatic inhibition.

In vitro, auto-PARylation of PARP2 correlates with its release from DNA (24,45,46). To determine whether the lack of auto-PARylation might explain the inhibitor-induced trapping of PARP2 in *PARP1/2* DKO cells, we generated GFP-PARP2-E545A and GFP-PARP2-H415A with alanine substitutions in the conserved H-Y-E catalytic triad of PARP2 (25–27). As a control, we determined the impact of E to A and H to A mutations on the release of PARP2 from phosphorylated nicked DNA substrate *in vitro*. Purified HA and EA mutants of PARP2 fold properly, as measured by CD spectroscopy (Supplementary Figure S3A). While NAD^+^ triggers auto-PARylation and release of WT PARP2 from DNA, neither the EA nor HA mutation can be released from DNA measured by the fluorescence polarization assay, suggesting defects in an auto-PARylation activity that are consistent with the role of these residues in catalyzing PARylation (Supplementary Figure S3B–D). Moreover, although HPF1 promotes auto-PARylation of WT PARP2, EA and HA mutants of PARP2 display no significant PARylation even in the presence of HPF1 (Supplementary Figure S3E).

**Figure 4.**
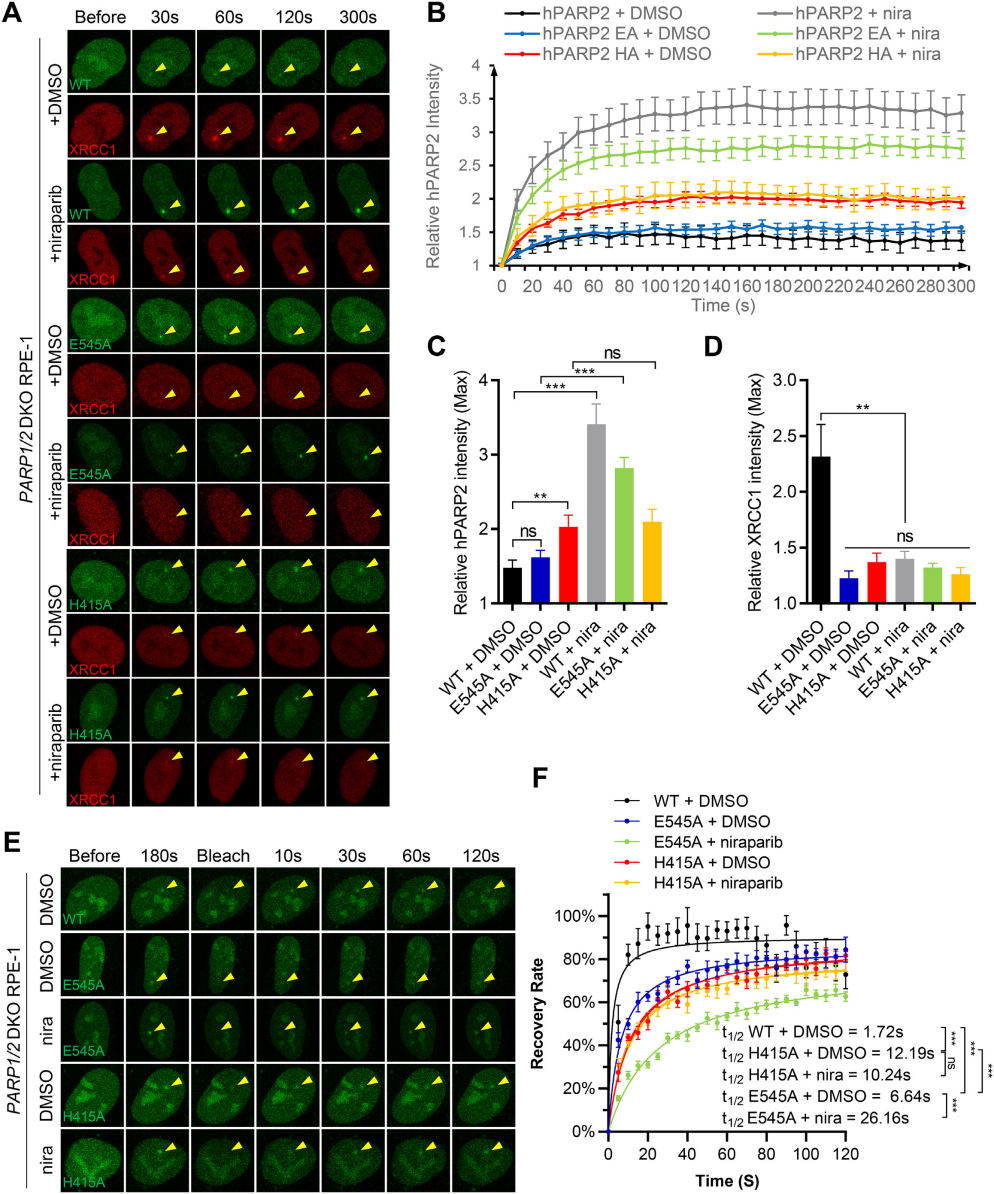
Catalytically inactive PARP2 can also be trapped by niraparib. (**A**) Representative images of GFP-PARP2 WT, E545A, H415A and mRFP-XRCC1, (**B**) the relative intensity kinetics of PARP2 WT, E545A and H415A at DNA damage site in *PARP1/2* DKO RPE-1 cells in the presence and absence of niraparib. (C and D) The maximal relative intensity of (**C**) GFP-PARP2 WT, E545A and H415A, and (**D**) mRFP-XRCC1. *P* value was calculated using the two-tailed unpaired Student's t-test. ns, *P* > 0.05; **P < 0.01; ***P < 0.001. (**E**) Representative images and (**F**) calculated FRAP recovery curves for GFP-PARP2 WT, E545A and H415A in *PARP1/2* DKO RPE-1 cells in the presence and absence of niraparib. *t*_1/2_ = 1.7 ± 0.9 s, *B*_max_= 90.4 ± 3.3% for WT + DMSO; *t*_1/2_ = 6.6 ± 1.4 s, *B*_max_= 85.8 ± 2.7% for E545A + DMSO; *t*_1/2_ = 26.2 ± 3.9 s, *B*_max_= 78.1 ± 3.6% for E545A + niraparib; *t*_1/2_ = 12.2 ± 2.1 s, *B*_max_= 87.6 ± 3.1% for H415A + DMSO; *t*_1/2_ = 10.2 ± 2.1 s, *B*_max_= 80.8 ± 3.1% for H415A + niraparib. *P* value was calculated using the extra sum-of-square *F* test. ns, *P* > 0.05; ***P < 0.001. All the dots and bars represent means and SEM, respectively, from one representative experiment out of 2–4 with *n* > 8 cells each time with consistent results.

Next, we measured the kinetics of PARP2-H415A and PARP2-E545A foci in *PARP1/2* DKO cells *in vivo*. Both PARP2-H415A and PARP2-E545A form weak yet consistent foci in *PARP1/2* DKO cells, with the PARP2-H415A foci brighter than PARP2-WT and PARP2-E545A foci (Figure 4A–C). Niraparib treatment intensified the PARP2-E545A foci, but not PARP2-H415A foci in *PARP1/2* DKO cells (Figure 4A–C). Corresponding to their lack of PARylation activity *in vitro*, neither H415A nor E545A-PARP2 supported robust XRCC1 foci *in vivo* (Figure 4A, and D). Similar results were found using *Parp1/2* DKO iMEFs (Supplementary Figure S4A–D). Next, we examined the exchange of PARP2-E545A and H415A mutants via FRAP. The results showed that both PARP2-E545A and H415A mutants recovered efficiently after photobleaching, reaching ∼80% of pre-breach levels within 2 min. The recovery for both mutants appeared slower than the PARP2-WT (*t*_1/2_ = 1.7 ± 0.9 s), with PARP2-H415A (*t*_1/2_ = 12.2 ± 2.1 s, *P* < 0.0001) even slower than PARP2-E545A (*t*_1/2_ = 6.6 ± 1.4 s, *P* < 0.0001) (Figure 4E, F and Supplementary Figure S4E). Nevertheless, niraparib further delayed the exchange of PARP2-E545A (to *t*_1/2_ = 26.2 ± 3.9 s, *P* < 0.0001), but did not measurably affect the exchange of PARP-H415A (*t*_1/2_ = 10.2 ± 2.1 s, *P* = 0.24) (Figure 4E, F and Supplementary Figure S4E). Together, these results indicate that the lack of auto-PARylation alone cannot explain the stalling of PARP2 by niraparib and identified a role of PARP2 H415 in niraparib-induced trapping of PARP2. In this context, purified PARP2-HA mutant consistently display lower affinity to phosphorylated nick (pNick) DNA substrates than the WT PARP2 (Supplementary Figure S3F).

### The NTR domain is dispensable for niraparib-induced trapping of PARP2 *in vivo*

**Figure 5.**
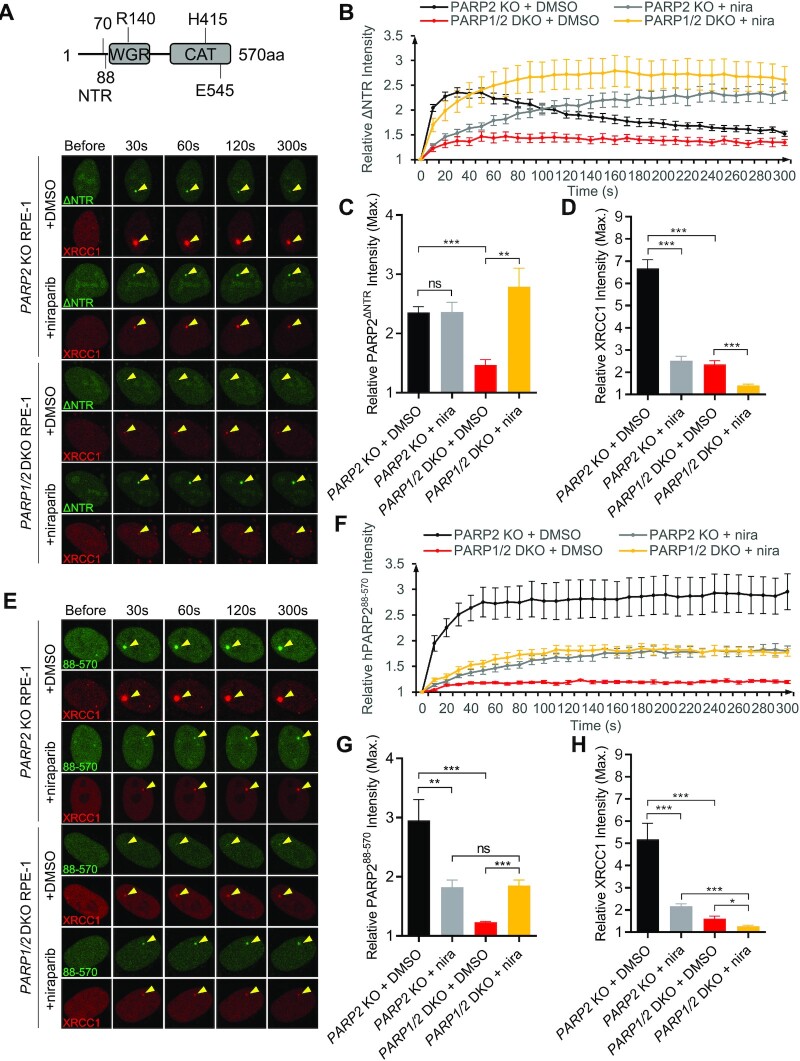
The NTR is largely dispensable for PARP2 recruitment and trapping by niraparib. (**A**) The schematic of PARP2 domain with aa number marked for human PARP2. Representative images of GFP-SV40NLS-ΔNTR-PARP2 and mRFP-XRCC1, and (**B**) the relative intensity kinetics of GFP-SV40NLS-ΔNTR-PARP2 at DNA damage sites in *PARP2* KO and *PARP1/2* DKO RPE-1 cells in the presence and absence of niraparib. (**C**, **D**) The maximal relative intensity of (C) GFP-SV40NLS-ΔNTR-PARP2 and (E) mRFP-XRCC1. (**E**) Representative images of GFP-SV40NLS-PARP2^88-570^ and mRFP-XRCC1, and (**F**) the relative intensity kinetics of GFP-SV40NLS- PARP2^88-570^ at DNA damage sites in *PARP2* KO and *PARP1/2* DKO RPE-1 cells in the presence and absence of niraparib. (G and H) The maximal relative intensity of (**G**) GFP-SV40NLS- PARP2^88-570^, and (**H**) mRFP-XRCC1. The dots and bars represent means and SEM, respectively, from one representative experiment out of 2–4 with *n* > 8 cells each time with consistent results. The two-tailed unpaired Student's *t*-test was used to calculate p-values. ns, *P* > 0.05; *P < 0.05; **P < 0.01; ***P < 0.001.

PARP2 binds to DNA, RNA, as well as PAR (15,47,48). Given that niraparib-induced trapping of PARP2 occurs even in PARP1-deficient cells in the presence of inhibitors, we hypothesized that PARP2 is trapped at the DNA breaks. Both the NTR and the WGR domain of PARP2 bind to DNA. NTR also binds to PAR (15). We therefore examined the impact of the NTR and WGR domains on PARP2 recruitment and niraparib induced trapping. We found that deletion of NTR (aa 1–70) alone of PARP2 (ΔNTR-PARP2) did not significantly affect PARP2 foci formation in PARP1-proficient cells (Figure 5A, B, and Supplementary Figure S5A–D for comparison with PARP1-WT), suggesting that NTR is dispensable for the PARP1 and PAR dependent recruitment of PARP2. Moreover, niraparib treatment delayed the ΔNTR-PARP2 foci in *PARP2* KO RPE-1 cells and intensified the weak ΔNTR-PARP2 foci in the *PARP1/2* DKO cells (Figure 5A–D). Overall, the kinetics of ΔNTR-PARP2 were quite similar to those of WT PARP2 (Figure 2A, B) regardless of the presence or absence of PARP1 or niraparib, suggesting that the NTR domain is largely dispensable for PARP2 recruitment and retention.

The exact amino acid definition of NTR (Supplementary Figure S5E) varies between different studies (15,24). While the NTR was defined as 1–70AA in most studies, 1–87AA of PARP2 was found to bind PAR and form damage induced foci (15). To ensure that the lack of impact of NTR is not due to insufficient deletion (1–70aa only), we made an extended ΔNTR-PARP2 (88–570AA), in which we deleted 1–87AA (∼17aa more than the ΔNTR-PARP2). Live-cell imaging studies suggest that ΔNTR-PARP2 (88–570AA) also forms robust foci (Figure 5E–G), which are reduced by PARP1-deficiency and enhanced by niraparib treatment (Supplementary Figure S1H and Figure 4C). Moreover, the foci formed by the large NTR deletion are persistent through the course of the study (5 min) in PARP1-proficient cells (Figure 5E and F). Finally, ΔNTR-PARP2 (large) formed significantly weaker foci in *PARP1/2* DKO cells than PARP2 WT and ΔNTR-PARP2 (small) both with and without niraparib, suggesting the additional 17 aa deletion in the unstructured NTR link region might compromise DNA binding (Figure 5E–G and Supplementary Figure S5A–C). Nevertheless, niraparib treatment enhanced ΔNTR-PARP2 (large) foci in *PARP1/2* DKO cells, suggesting that another domain beyond NTR might contribute to DNA binding and niraparib mediated trapping of PARP2.

### The WGR domain is essential for PARP1-independent recruitment and retention of PARP2 on DNA

**Figure 6.**
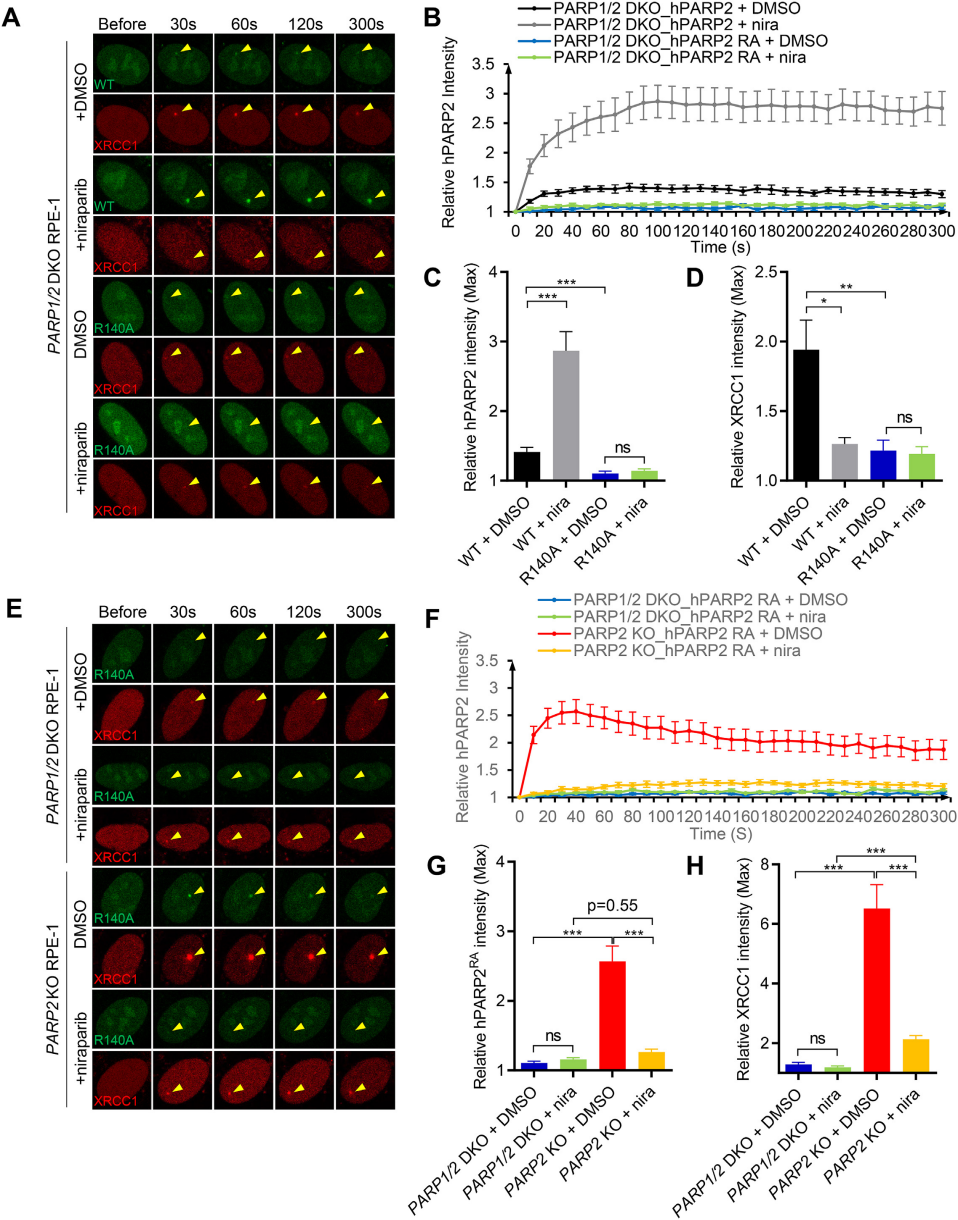
The R140A mutation in the WGR domain abrogates PARP1-independent recruitment and trapping of PARP2. (**A**) Representative images of GFP-PARP2 WT, R140A, and mRFP-XRCC1 and (**B**) the relative intensity kinetics of PARP2 WT and R140A at DNA damage sites in *PARP1/2* DKO RPE-1 cells in the presence and absence of niraparib. (C, D) The maximal relative intensity of (**C**) GFP-PARP2 WT and R140A, and (**D**) mRFP-XRCC1. (**E**) Representative images of GFP-PARP2-R140A and mRFP-XRCC1 and (**F**) the relative intensity kinetics of PARP2-R140A at DNA damage sites in *PARP2* KO and *PARP1/2* DKO RPE-1 cells in the presence and absence of niraparib. (G, H) The maximal relative intensity of (**G**) GFP-PARP2-R140A, and (**H**) mRFP-XRCC1. The dots and bars represent means and SEM, respectively, from one representative experiment out of 2–4 with *n* > 8 cells each time with consistent results. The two-tailed unpaired Student's *t*-test was used to calculate *P*-values. ns, *P* > 0.05; *P < 0.05; ***P < 0.001.

**Figure 7.**
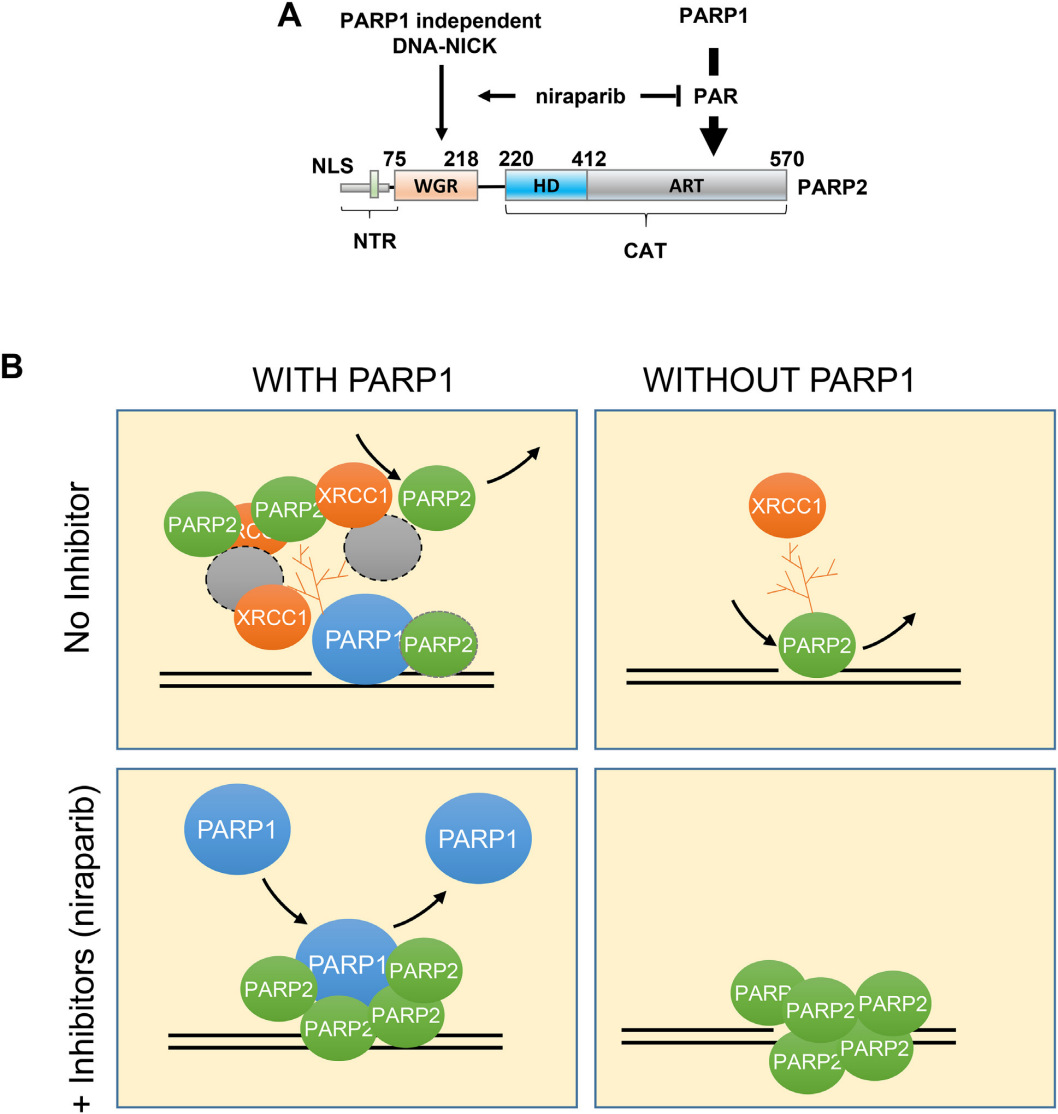
Niraparib converts the predominantly PARP1-dependent recruitment of PARP2 to direct trapping of PARP2 at the DNA. (**A**) The panel shows the two modes of PARP2 foci formation. On the right is the PARP1-dependent and PAR-dependent mode. On the left is the PARP1-independent and direct DNA binding mode via the WGR domain. PARP inhibitor, niraparib, suppresses PAR formation and attenuates PARP1-dependent PARP2 foci, while enhancing the PARP1-independent PARP2 foci by trapping PARP2 on DNA. (**B**)The diagram shows the four scenarios—with or without PARP1 (left and right) and with or without PARP inhibitors (*e.g*. niraparib, upper row, or lower row). Upper-left: in the presence of PARP1, the majority of PARP2 is recruited to the foci through a PARP1-dependent and PAR-dependent manner via either direct interaction with PAR or via another PAR binding protein. Upper-right: in the absence of PARP1, PARP2 foci intensity is reduced significantly (∼1/4 of the levels of those with PARP1, depicted by one PARP2 icon) and is mediated by direct interaction between the WGR motif of PARP2 and DNA. Lower-right: in PARP1-deficient cells, PARP inhibitor (niraparib) traps PARP2 at the DNA damage sites to form strong foci (depicted by 5 PARP2 icons). Lower-left: in PARP inhibitor-treated cells with PARP1, the overall intensity of PARP2 foci does NOT change (still 4 PARP2 icons). But they are now primarily made up of direct interaction between PARP2 and DNA since little or no PAR is there. In this case, PARP1 might be continuously recruited and compete with PARP2 for DNA binding.

Next, we tested whether the WGR domain, implicated in DNA binding and the allosteric activation of PARP2, is required for niraparib dependent trapping of PARP2. Structural analyses have identified a critical role for the R140 residue within the WGR domain for PARP2 DNA binding (49,50). *In vivo*, we found that PARP2-R140A failed to form foci in *PARP1/2* DKO cells and cannot be ‘trapped’ by niraparib (Figure 6A–C). XRCC1 foci were also very dim in the PARP2-R140A transfected *PARP1/2* DKO cells (Figure 6D), consistent with the lack of significant PARylation activity from PARP2-R140A. With this mutant PARP2 lacking DNA binding in hand, we sought to ask how much direct DNA binding of PARP2 contributes to PARP2 foci formation in PARP1 proficient cells. In contrast to the complete lack of foci in *PARP1/2* DKO cells, PARP2-R140A formed robust and nearly normal foci in PARP1-proficient cells (*PARP2* KO) (Figure 6E–H). The kinetics and max intensity of PARP2-R140A foci in *PARP2* KO cells are comparable to those of PARP2-WT (Supplementary Figure S6A–D), indicating that R140, and by extension, DNA binding, is dispensable for PARP1-dependent recruitment of PARP2. Moreover, we found that in PARP1-proficient cells, niraparib completely abolished PARP2-R140A foci (Figure 6E–G), suggesting that PAR generated by PARP1, but not PARP1 protein itself, is mainly responsible for the PARP1-dependent recruitment of PARP2. Consistent with this mode of action, PARP2-R140A cannot be trapped at the chromatin by niraparib (Supplementary Figure S6E) and ectopically expressed PARP2-R140A also failed to alter the niraparib sensitivity of *PARP1/2* DKO cells (Supplementary Figure S6F and G). The findings therefore suggest that the delayed, yet robust, accumulation of WT-PARP2 in niraparib treated PARP1-proficient cells is consistent with WGR-dependent trapping of PARP2 on DNA and abolished by the R140A mutation. Taken together, our findings support a model in which PARP2 is recruited to DNA damage sites via both a PARP1 dependent and PARP1 independent modes (Figure 7A) where niraparib prevents PARylation, thereby suppressing PARP1-dependent recruitment of PARP2, while enhancing the PARP1-independent direct binding of PARP2 to DNA (Figure 7A). As a result, niraparib alters the nature of PARP2 at the foci by converting the predominantly PARP1 and PAR dependent recruitment of PARP2 to a DNA-dependent and PARP1-independent physical trapping of PARP2 (Figure 7B).

## DISCUSSION

Purified PARP2 can bind to PAR and RNA, and can be activated by DNA (15,24,28,51). The relative contribution of these modes of binding to the recruitment and retention of PARP2 *in vivo* and in the presence of PARP inhibitors remains elusive. Using quantitative live-cell imaging, we showed that PARP2 can be recruited to DNA damage sites through both a PARP1-dependent (predominantly) and a PARP1-independent mechanism. Loss of endogenous PARP1 and its PARylation activity markedly attenuate PARP2 foci (Figure 1A and D). PARP2-R140A, lacking DNA binding, can still form robust foci in PARP1 expressing cells (Figure 6E–G), suggesting the majority of PARP2 at the DNA damage foci is formed via the PARP1-dependent mechanism. Niraparib nearly completely abolishes the PARP2-R140A foci in PARP1-proficient cells (Figure 6E–G), indicating it is the PAR generated by PARP1, and not PARP1 protein itself, that recruits PARP2. Although the NTR domain of PARP2 can bind PAR *in vitro* and *in vivo* (15), both ΔNTR-PARP2 (del aa 1–70 and aa 1–87) mutants form robust DNA damage foci in PARP1 proficient cells, suggesting another domain of PARP2 is sufficient to mediate PARP1 and PAR-dependent recruitment of PARP2. We consider two possibilities. For one, PARP2 preferentially generates a branched PAR chain and can interact with the PAR chain terminus as an enzyme-substrate duo. Prior studies have implicated the CAT-domain of PARP2 in foci formation (24). Alternatively, PARP2 might form a complex with other PAR binding proteins (*e.g*., XRCC1 or PCNA) (6,52,53), which bring PARP2 to the foci in a PAR-dependent manner.

On the other hand, in PARP1-deficient cells, PARP2 formed moderate, yet consistent, foci that were markedly enhanced by niraparib and abrogated by the R140A mutation. Given the R140A mutation abolishes DNA binding of PARP2 (54) and PARP inhibitor caused trapping of PARP2 on DNA is weakened by the H415A mutation in the catalytic domain, these findings suggest a model in which niraparib interacts with the catalytic domain and allosterically prevent PARP2 release from DNA via the WGR domain. It further suggests that the PARP1-independent PARP2 foci are mediated by direct interaction between the PARP2 WGR domain and DNA (Figure 7A). Indeed, two other mutations in the WGR domain—N116A and Y188F—also compromise PARP2 foci formation *in vivo* (24). We also note that the PARP2 foci in niraparib treated *PARP1/2* DKO cells are brighter than those in PARP1 proficient (i.e. *PARP2* KO) cells, consistent with a model where endogenous PARP1 competes with the WGR domain of PARP2 for DNA binding. Despite the ability for NTR to bind to DNA and PAR, we, like others (24), found that deletion of NTR has at most a moderate impact on PARP2 foci formation regardless of PARP1 status (Supplementary Figure S5). We noted that PARP2-ΔNTR (large, Δaa1-87) produces potentially weaker PARP2 foci in *PARP1/2* DKO cells than the PARP2-ΔNTR (Δaa1–70), highlighting a role for aa 70–87 of the unstructured NTR region for DNA binding. Two isoforms of PARP2 were noted in UniProt (Entry: Q9UGN5). Isoform 2 was used here and in prior studies (15,24,51). Isoform 1 has an additional 13 aa after aa 67 of isoform 2 (Supplementary Figure S5E). Although the long isoform has been used for several structural studies, the NTR is not resolved, presumably due to high-degree of freedom. Whether differential expression of the long versus short isoforms might affect the relative contribution of PARP2 versus PARP1 to DNA damage-induced PARylation remains unknown.

Nearly a decade has passed since the Pommier group first reported that PARP inhibitors trap PARP2 on chromatin after DNA damage (17), but the nature of PARP2 trapping remains elusive. Recently, Blessing *et al.* reported that three PARP inhibitors—veliparib, olaparib and talazoparib—delayed the recruitment and release, and reduced the maximal enrichment of PARP2 at DNA damage sites in U2OS cells (18). Among these, veliparib is not known to trap PARP1 or PARP2. Consistent with their finding, we found that PAR generated by PARP1, not PARP1 itself, promotes PARP2 recruitment. In the PARP1 proficient RPE-1 cells and iMEFs, niraparib delayed the initial recruitment of PARP2. Notably, at the later timepoints (∼2 min in murine *Parp2* KO iMEFs, Figure 2B and ∼3 min in *PARP2* KO human RPE1 cells, Supplementary Figure S1G), the maximal intensity of PARP2 foci reached a level near those in the untreated cells. Given XRCC1 foci are largely abolished in niraparib treated cells, this result indicates that PARP2 can also be recruited to the DNA damage sites through a PARP1 and PAR-independent mechanism. In this context, niraparib and talazoparib markedly enhanced PARP2 foci in *PARP1/2* DKO and decreased PARP2 exchange on DNA measured by FRAP (Figure 3). PARP2 is trapped on DNA since the R140A mutation in the WGR domain completely abolished the trapping (Figure 6). The maximal intensity of PARP2 foci in niraparib treated *PARP2* KO (PARP1 proficient) cells are consistently lower than those in *PARP1/2* DKO cells, suggesting that endogenous PARP1 might compete with PARP2-DNA interaction (Supplementary Figure S1F–H). In this context, it takes longer for PARP2 foci to accumulate in PARP1 proficient human cells than in PARP1 proficient mouse cells, an observation that is consistent with the higher levels of endogenous PARP1 in human cells than in murine cells. Alternatively, the residual PARP1 mediated PARylation activity might recruit other PAR-binding factors, like ALC1, to promote PARP2 release (18). Despite nearly equally efficient PAR-inhibition measured by reduced XRCC1 foci (Figure 2E and H), three different PARP inhibitors exhibit different PARP2-trapping potentials: niraparib = talazoparib > olaparib (Figure 2E–G). Niraparib also enhanced the foci formed by the auto-PARylation deficient PARP2-E545A (Figure 4). These observations suggest that niraparib and talazoparib allosterically lock PARP2 at the DNA damage sites via the WGR domain in a mechanism that is largely independent of auto-PARylation. While both the HA and EA mutants of PARP2 are inactive (Supplementary Figure S3), the HA mutation is less responsive to the niraparib-induced trapping (Figure 4), potentially due to weaker DNA binding by the HA-mutant (Supplementary Figure S3E and F). Alternatively, the H415A mutation might compromise the inhibitor-induced allosteric locking.

Using live-cell imaging and FRAP, our study revealed the mechanism underlying PARP2 trapping that is distinct from PARP1. In the case of PARP1, PARP inhibitors delay DNA repair without affecting PARP1 exchange, to cause continuous recruitment and exchange of PARP1 and persistent foci (16) (Figure 7). In the case of PARP2, PARP inhibitor changes the DNA damage-induced PARP2 foci from a predominantly PARP1 and PAR-dependent gathering of PARP2 to a relatively stable interaction of PARP2 with DNA via the WGR domain. Given PARP1 and PARP2 compete with each other for DNA binding, PARP inhibitors might increase the relative abundance of PARP2 at the DNA lesion. Despite the difference, the H415A mutation of PARP2 and the H862A mutation of PARP1 (16) both led to resistance to niraparib induced ‘trapping’, highlighting a conserved role of the H in the H-Y-E triad of PARP1 and 2 in the inhibitor response. Detailed structural analyses will help us elucidate the exact movement underpinning this allosteric trapping and guide the development of selective PARP1 or PARP2 inhibitors in the future (55).

## Supplementary Material

gkac188_Supplemental_File
