# Supplementary material for: PARP inhibitors trap PARP2 and alter the mode of recruitment of PARP2 at DNA damage sites
Source: Nucleic Acids Res. 2022 Mar 29;50(7):3958–73. doi: 10.1093/nar/gkac188 (PMC9023293; doi:10.1093/nar/gkac188)
Supplement: gkac188_Supplemental_File [file gkac188_supplemental_file.pdf]

Supplementary Figure Legends

Figure S1. PARP1 regulates PARP2 recruitment and retention in human RPE-1 cells
(A) The targeting scheme of Parp1 conditional mice and southern blot of successfully targeted ES clones. The schematic diagram represents the murine Parp1 locus (top), targeting vector (2nd row), targeted allele (3rd row), and the neo-deleted allele (Parp1^{C}, bottom). The southern probe is marked as thick black lines and the exons and FRT sequences are shown as black solid boxes and open triangles, respectively. Restriction site designation: ScaI. The map is not drawn to scale. (B) Western blot of endogenous Parp1 and Parp2 proteins in WT, *Parp1* KO, *Parp2* KO, and *Parp1/2* DKO iMEF cells. (C) The kinetics of mRFP-XRCC1 foci (relative intensity) in *Parp2* KO and *Parp1/2* DKO iMEF cells in the presence and absence of niraparib. (D) The normalized PARP2 intensity at DNA damage sites in *Parp2* KO and *Parp1/2* DKO iMEFs in the presence and absence of niraparib. (E) Western blot of endogenous PARP1 and PARP2 levels in wild type, *PARP1* KO, *PARP2* KO, and *PARP1/2* DKO RPE-1 cells. (F) Representative images of GFP-PARP2 and mRFP-XRCC1, and (G) the relative intensity kinetics of GFP-PARP2 at DNA damage sites in *PARP2* KO and *PARP1/2* DKO RPE-1 cells in the presence and absence of niraparib. (H) The maximal relative intensity of GFP-PARP2. The dots and bars represent means and SEM, respectively, from one representative experiment out of 2–4 with n > 8 cells each time with consistent results. The two-tailed unpaired Student's t-test. ns, p > 0.05; **p < 0.01; ***p < 0.001.

Figure S2. The time of photobleaching does not affect recovery kinetics and niraparib also enhances PARP2 foci from 8-MOP primed cells.
(A) The relative intensity of GFP-PARP2 before photobleaching at 3 min post-irradiation in *PARP1/2* DKO RPE-1 cells in the presence and absence of PARP inhibitors. (B) Calculated FRAP recovery curves for GFP-PARP2 foci photo-bleached at the 60s and 180s post-irradiation in *PARP1/2* DKO RPE-1 cells without PARP inhibitors. $t_{1/2}$ = 1.3 ± 0.9s, B_{max} 88.7 ± 3.3% for 60s; $t_{1/2}$ = 1.6 ± 0.7s, B_{max} = 90.3 ± 2.5% for 180s. *P*-value was calculated based on the extra sum-of-square *F* test. ns, p > 0.05. (C) Representative images of GFP-PARP2 and mRFP-XRCC1 upon 60% 405 nm laser-induced micro-irradiation in *PARP1/2* DKO RPE-1 cells in the presence and absence of 8-MOP. (D) Representative images of GFP-PARP2 and mRFP-XRCC1, and the relative intensity kinetics of (E) GFP-PARP2 and (F) mRFP-XRCC1 upon 20% 405nm laser-induced micro-irradiation in 8-MOP treated *PARP1/2* DKO RPE-1 cells in the presence and absence of niraparib. (G) The relative intensity of GFP-PARP2 before photo-bleaching at 3 min post-irradiation in 8-MOP treated *PARP1/2* DKO RPE-1 cells in the presence and absence of niraparib. (H and J) The maximal relative intensity of (H) GFP-PARP2, and (J) mRFP-XRCC1 in *PARP1* KO RPE-1 cells in the presence and absence of niraparib. (I) The relative intensity of GFP-PARP2 before photobleaching at 3 min post-irradiation in *PARP1* KO RPE-1 cells in the presence and absence of niraparib. The dots and bars represent means and SEM, respectively, from one representative experiment out of 2–4 with n > 8 cells each time with consistent results. The two-tailed unpaired Student's t-test. ***p < 0.001.

Figure S3 Trapping of PARP2 required the physical presence of niraparib in MEF cells.
(A) CD spectrometry measurement of purified WT and EA/HA mutant PARP2 (0.2 mg/mL) in 20 mM Potassium Phosphate (pH 7.5), 0.1 mM EDTA, 0.1 mM TCEP. All spectra are reported in Mean Residue Ellipticity. (B-D) Representative pNick release data for (B) human PARP2 WT, (C) murine Parp2-H404A, and (D) Parp2-E534A with NAD+. (E) Representative pNick release data for WT and mutant PARP2 in the presence of purified HPF1. (F) Binding affinity of WT-PARP2 and H404A-Parp2 to the pNick model DNA substrate. The calculated Kd were marked under the graph.

Figure S4 PARP2 trapping in niraparib treated iMEF cells does not correlate with PARP2 activity.
(A) Representative images of GFP-PARP2 WT, E545A, H415A, and mRFP-XRCC1 in *Parp1/2 DKO* iMEFs. (B) the relative intensity kinetics of PARP2 WT, E545A, and H415A at DNA damage sites in *PARP1/2* DKO RPE-1 cells in the presence and absence of niraparib. (C and D) The maximal relative

intensity of (C) GFP-PARP2 WT, E545A and H415A, and (D) mRFP-XRCC1. (E) The relative intensity of GFP-PARP2-E545A and -H415A before photo-bleaching at 3 min post-irradiation in *PARP1/2* DKO RPE-1 cells in the presence and absence of niraparib. The dots and bars represent means and SEM, respectively, from one representative experiment out of 2–4 with n > 8 cells each time with consistent results. The two-tailed unpaired Student's t-test. ns, p > 0.05; *p < 0.05; **p < 0.01; ***p < 0.001

Figure S5 NTR does not affect PARP2 foci formation.
(A) Representative images of GFP-PARP2 WT, PARP2$^{\Delta NTR}$ (Δ1-70aa), PARP2$^{88\text{-}570,}$ and mRFP-XRCC1 in *PARP1/2* DKO cells. (B) The relative intensity kinetics of GFP-PARP2 WT, PARP2$^{\Delta NTR,}$ and PARP2$^{88\text{-}570}$ at DNA damage sites in *PARP2* KO and *PARP1/2* DKO RPE-1 cells. (C and D) The maximal relative intensity of (C) GFP-PARP2 WT, PARP2$^{\Delta NTR}$ and PARP2$^{88\text{-}570}$, and (D) mRFP-XRCC1. (E) Alignment of the N-terminal region of human (isoform 1 and 2) and mouse PARP2 from the Uniprot database. The aa in red are those that differ in the small and large NTR deletion.

Figure S6 Niraparib abolished PARP2 R140A foci, but not PARP2 WT foci in PARP1 proficient RPE-1 cells.
(A) Representative images of GFP-PARP2 WT, R140A, and mRFP-XRCC1 in PARP1 proficient RPE1 cells. (B) The relative intensity kinetics of GFP-PARP2 WT and R140A at DNA damage sites in *PARP2* KO (PARP1-proficient) RPE-1 cells in the presence and absence of niraparib. (C and D) The maximal relative intensity of (C) GFP-PARP2 WT and R140A, and (D) mRFP-XRCC1. The dots and bars represent means and SEM, respectively, from one representative experiment out of 2–4 with n > 8 cells each time with consistent results. The two-tailed unpaired Student's t-test. ns, p > 0.05; *p < 0.05; **p < 0.01; ***p < 0.001. (E) Soluble and chromatin fraction of *PARP1* KO RPE-1 cells and *PARP1/2* DKO RPE-1 cells ectopically expressing PARP2-R140A that are treated with MMS (0.1 mg/mL, 1 hr) and/or niraparib (1 µM, 1 hr). (F) Flow cytometry analyses of the frequency of hCD8 positive *PARP1/2* DKO + empty-hCD8 and *PARP1/2* DKO + hCD8/PARP2^{RA} RPE-1 cells at day 0 and day 6 of niraparib sensitivity assay. (G) Niraparib sensitivity assay for *WT*, *PARP1* KO, *PARP1/2* DKO + hCD8 and *PARP1/2* DKO + hCD8/PARP2^{RA}. The dots and error bars represent means and SEM, respectively, from one representative experiment out of 3 consistent biological repeats with triplicate samples per experiment. *P* value of IC50 was calculated using the extra sum-of-square *F* test. ns, p > 0.05; ***p < 0.001.

Figure S1

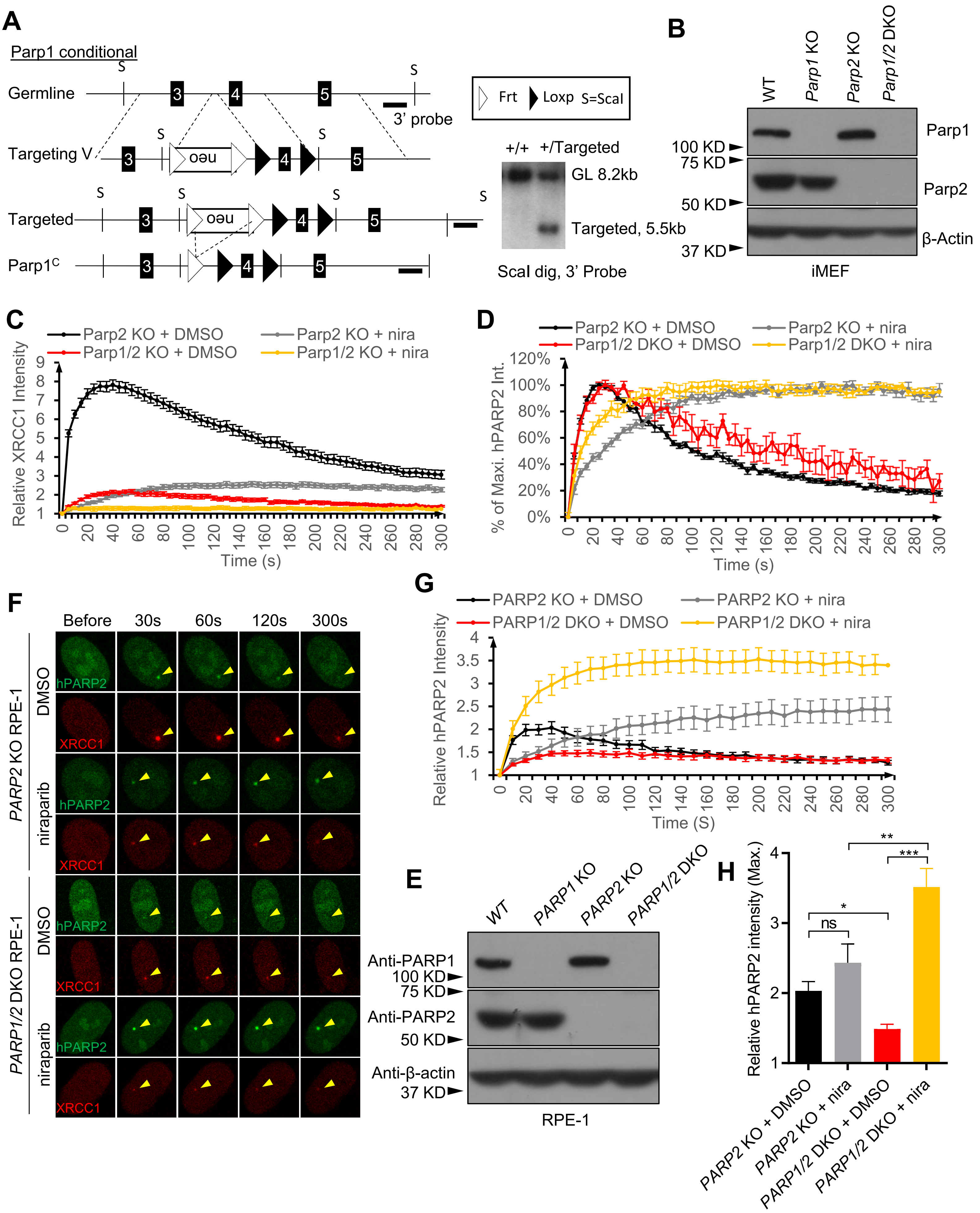

A
Parp1 conditional
Germline
3' probe
Targeting V
neo
Targeted
Parp1C
Frt
Loxp
S=ScaI
+/+
+/Targeted
GL 8.2kb
Targeted, 5.5kb
ScaI dig, 3' Probe
B
WT
Parp1 KO
Parp2 KO
Parp1/2 DKO
Parp1
Parp2
β-Actin
100 KD
75 KD
50 KD
37 KD
iMEF
C
Parp2 KO + DMSO
Parp2 KO + nira
Parp1/2 KO + DMSO
Parp1/2 KO + nira
Relative XRCC1 Intensity
Time (s)
D
Parp2 KO + DMSO
Parp2 KO + nira
Parp1/2 DKO + DMSO
Parp1/2 DKO + nira
% of Maxi. hPARP2 Int.
Time (s)
F
Before
30s
60s
120s
300s
PARP2 KO RPE-1
PARP1/2 DKO RPE-1
DMSO
niraparib
hPARP2
XRCC1
G
PARP2 KO + DMSO
PARP2 KO + nira
PARP1/2 DKO + DMSO
PARP1/2 DKO + nira
Relative hPARP2 Intensity
Time (S)
E
WT
PARP1 KO
PARP2 KO
PARP1/2 DKO
Anti-PARP1
Anti-PARP2
Anti-β-actin
100 KD
75 KD
50 KD
37 KD
RPE-1
H
Relative hPARP2 intensity (Max.)
ns
*
**
***
PARP2 KO + DMSO
PARP2 KO + nira
PARP1/2 DKO + DMSO
PARP1/2 DKO + nira

Figure S2

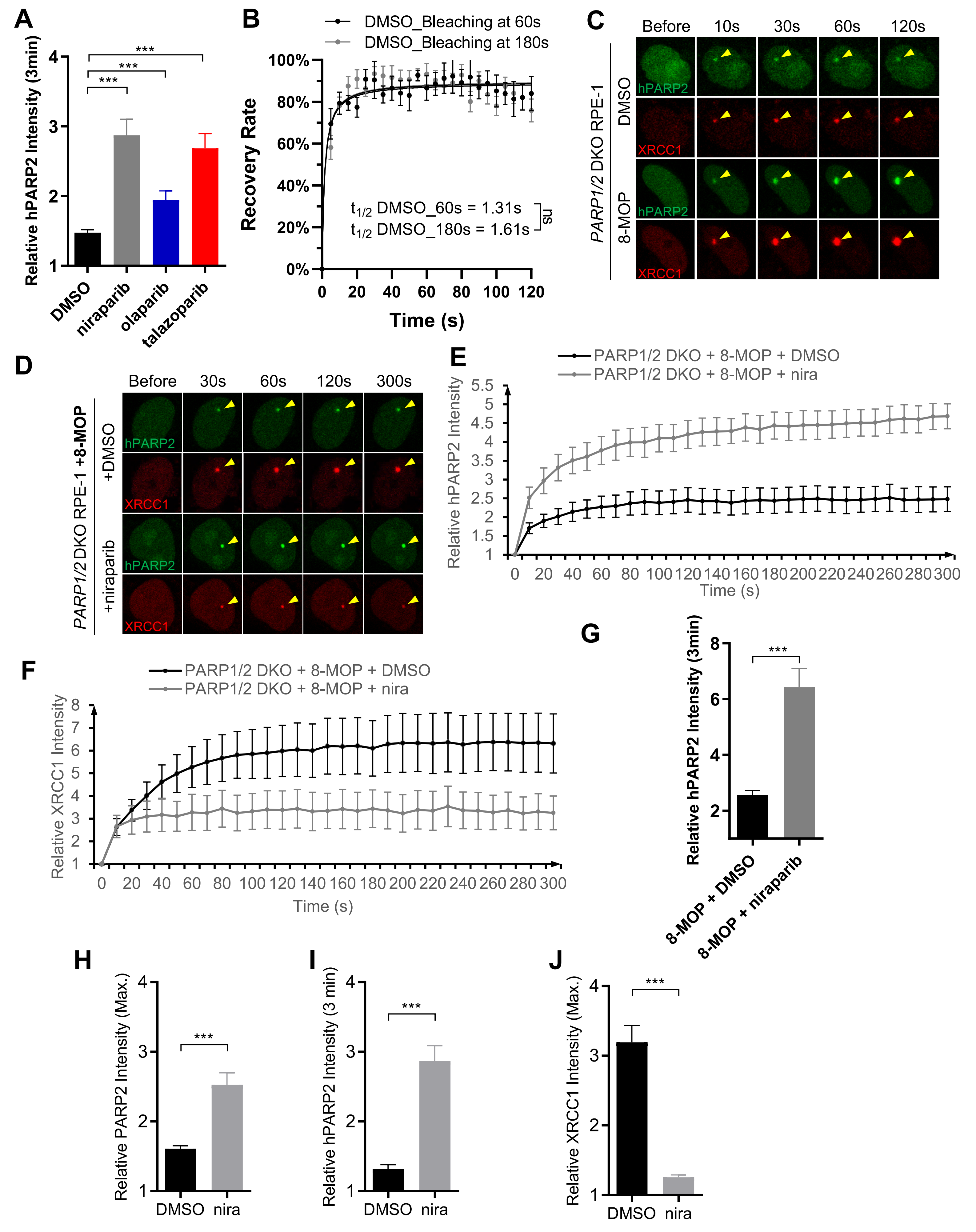
A
Relative hPARP2 Intensity (3min)
***
DMSO
niraparib
olaparib
talazoparib
B
DMSO_Bleaching at 60s
DMSO_Bleaching at 180s
Recovery Rate
$t_{1/2}$ DMSO_60s = 1.31s
$t_{1/2}$ DMSO_180s = 1.61s
ns
Time (s)
C
Before
10s
30s
60s
120s
PARP1/2 DKO RPE-1
DMSO
8-MOP
hPARP2
XRCC1
D
Before
30s
60s
120s
300s
PARP1/2 DKO RPE-1 +8-MOP
+DMSO
+niraparib
hPARP2
XRCC1
E
PARP1/2 DKO + 8-MOP + DMSO
PARP1/2 DKO + 8-MOP + nira
Relative hPARP2 Intensity
Time (s)
F
PARP1/2 DKO + 8-MOP + DMSO
PARP1/2 DKO + 8-MOP + nira
Relative XRCC1 Intensity
Time (s)
G
Relative hPARP2 Intensity (3min)
***
8-MOP + DMSO
8-MOP + niraparib
H
Relative PARP2 Intensity (Max.)
***
DMSO
nira
I
Relative hPARP2 Intensity (3 min)
***
DMSO
nira
J
Relative XRCC1 Intensity (Max.)
***
DMSO
nira

Figure S3

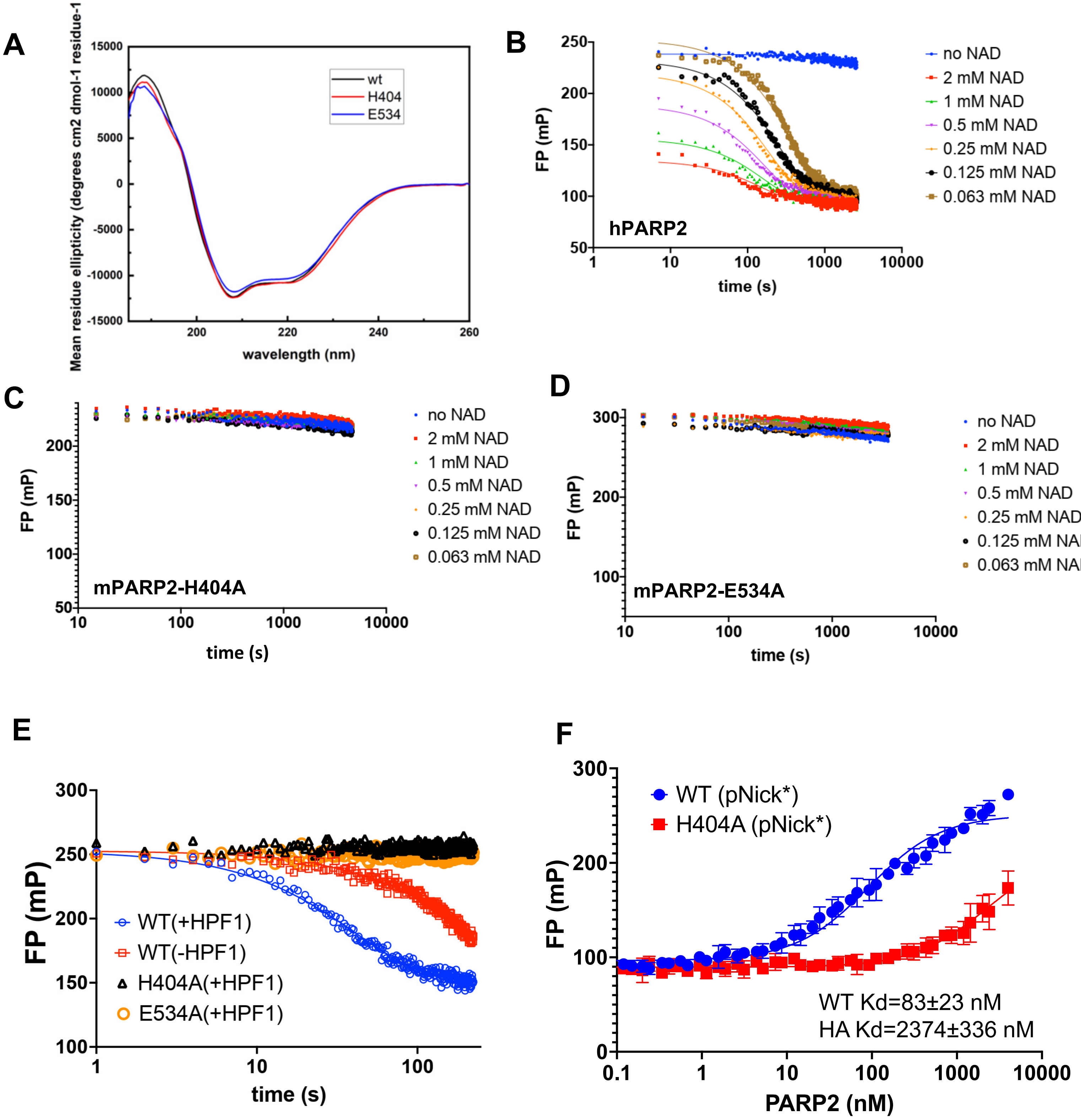

A
Mean residue ellipticity (degrees cm2 dmol-1 residue-1
wavelength (nm)
wt
H404
E534
B
FP (mP)
time (s)
hPARP2
no NAD
2 mM NAD
1 mM NAD
0.5 mM NAD
0.25 mM NAD
0.125 mM NAD
0.063 mM NAD
C
FP (mP)
time (s)
mPARP2-H404A
no NAD
2 mM NAD
1 mM NAD
0.5 mM NAD
0.25 mM NAD
0.125 mM NAD
0.063 mM NAD
D
FP (mP)
time (s)
mPARP2-E534A
no NAD
2 mM NAD
1 mM NAD
0.5 mM NAD
0.25 mM NAD
0.125 mM NAD
0.063 mM NAD
E
FP (mP)
time (s)
WT(+HPF1)
WT(-HPF1)
H404A(+HPF1)
E534A(+HPF1)
F
FP (mP)
PARP2 (nM)
WT (pNick*)
H404A (pNick*)
WT Kd=83±23 nM
HA Kd=2374±336 nM

Figure S4

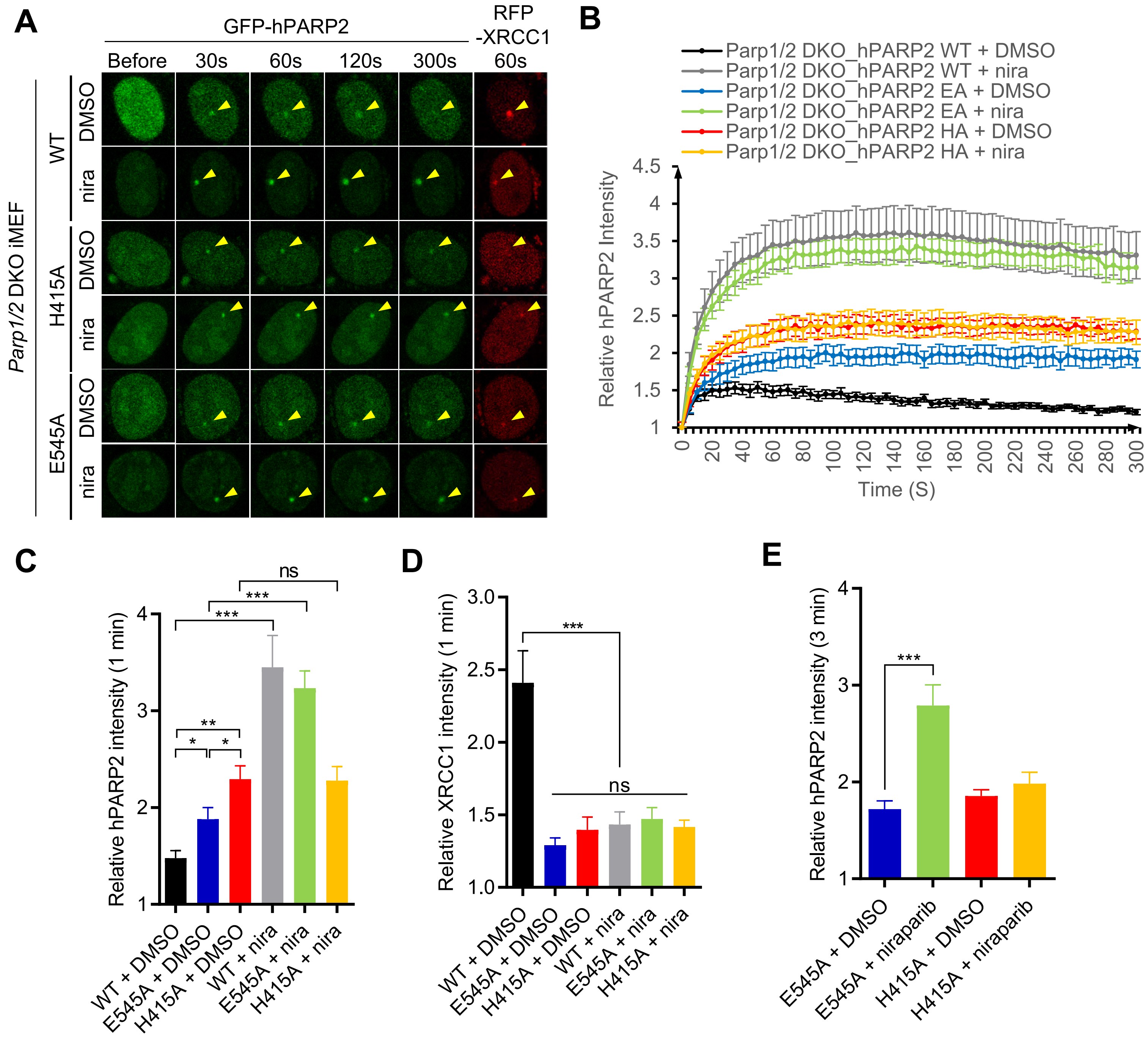

A
GFP-hPARP2
RFP -XRCC1
Before
30s
60s
120s
300s
60s
Parp1/2 DKO iMEF
WT
H415A
E545A
DMSO
nira
B
Parp1/2 DKO_hPARP2 WT + DMSO
Parp1/2 DKO_hPARP2 WT + nira
Parp1/2 DKO_hPARP2 EA + DMSO
Parp1/2 DKO_hPARP2 EA + nira
Parp1/2 DKO_hPARP2 HA + DMSO
Parp1/2 DKO_hPARP2 HA + nira
Relative hPARP2 Intensity
Time (S)
C
Relative hPARP2 intensity (1 min)
WT + DMSO
E545A + DMSO
H415A + DMSO
WT + nira
E545A + nira
H415A + nira
ns
***
**
*
D
Relative XRCC1 intensity (1 min)
WT + DMSO
E545A + DMSO
H415A + DMSO
WT + nira
E545A + nira
H415A + nira
E
Relative hPARP2 intensity (3 min)
E545A + DMSO
E545A + niraparib
H415A + DMSO
H415A + niraparib

Figure S5

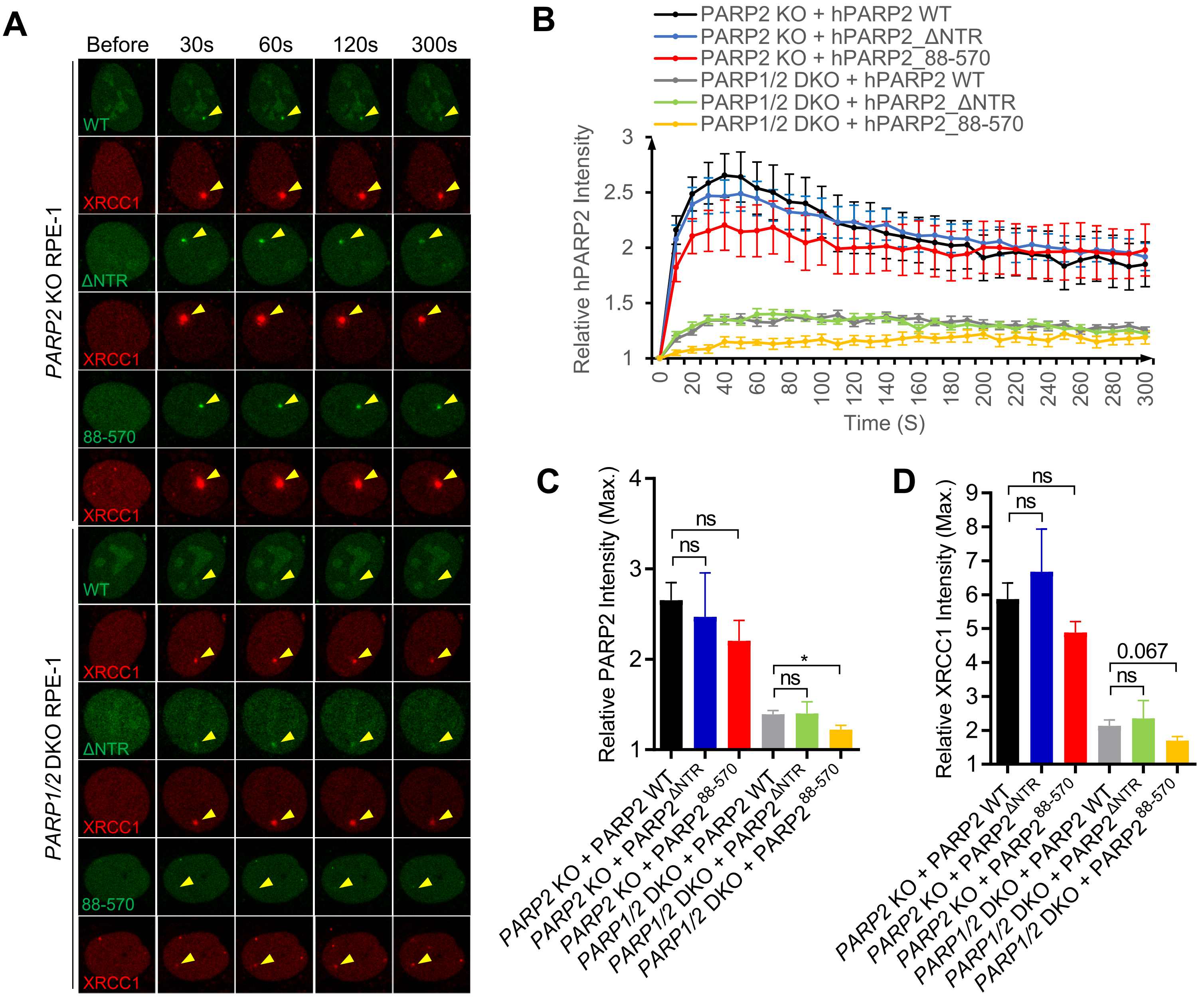

E

```
hPARP2(iso.1)  MAARRRRSTGGGRARALNESKRVNNGNTAPEDSSP-AKKTRRCQRQESKKMPVAGGKANK 59
hPARP2(iso.2)  MAARRRRSTGGGRARALNESKRVNNGNTAPEDSSP-AKKTRRCQRQESKKMPVAGGKANK 59
mPARP2         MAPRRQR--SGSGRRVLNEAKKVDNGNKATEDDSPPGKKMRTCQRK----GPMAGGKDAD 54
               ** **:*   .*.  *.***:*:*:***.*  **.**  .**  *  ***:     *:****   .

hPARP2(iso.1)  DRTEDKQDGMPGRSWASKRVSESVKALLLKGKAPVDPECTAKVGKAHVYCEGNDVYDVML 119
hPARP2(iso.2)  DRTEDKQD-------------ESVKALLLKGKAPVDPECTAKVGKAHVYCEGNDVYDVML 106
mPARP2         -RT---------------KDNRDSVKTLLLKGKAPVDPECAAKLGKAHVYCEGDDVYDVML 99
                **                    :***:*************:**:*********:*******
```

aa 71-87 different in long vs short NTR deletion

Figure S6

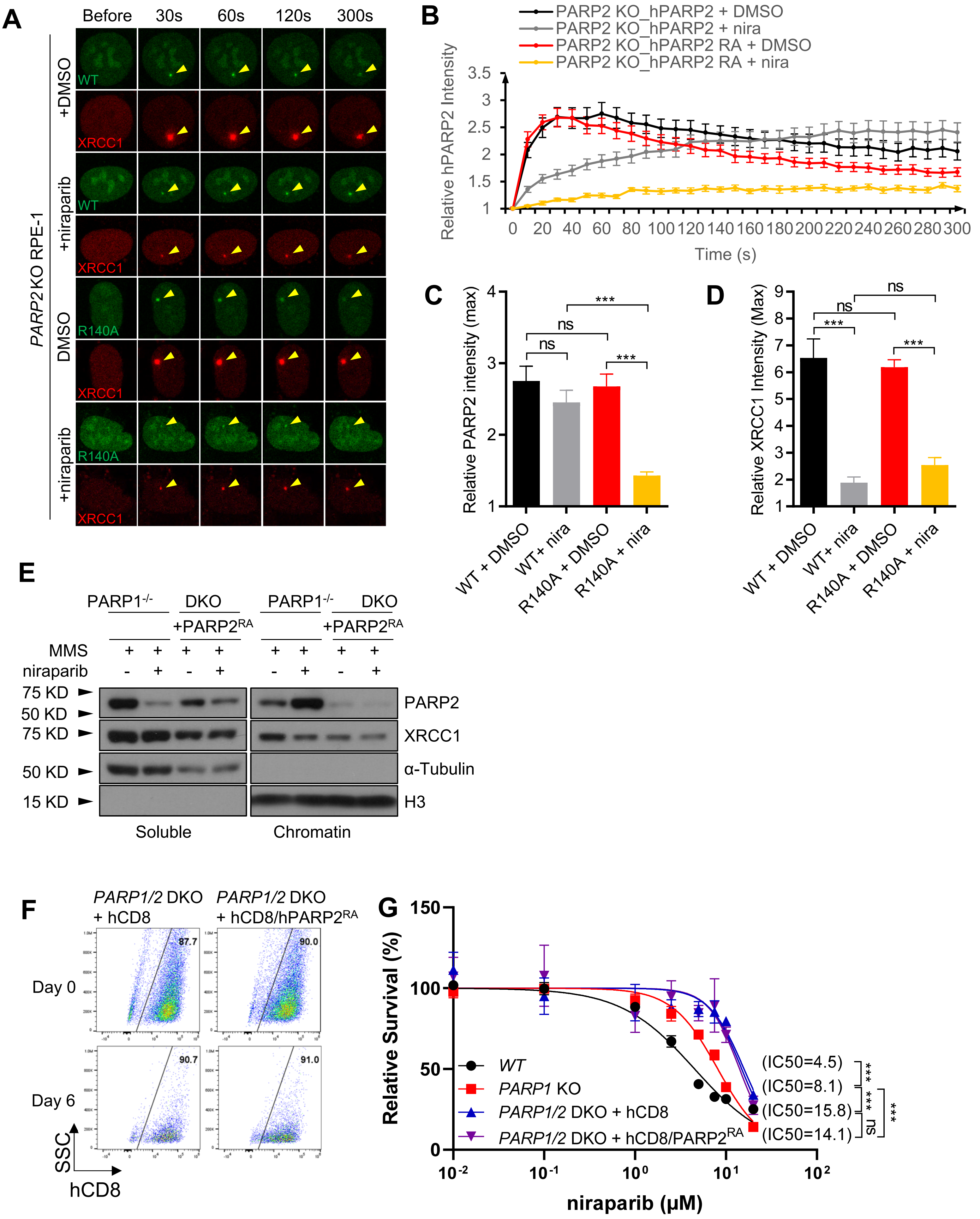
A
Before
30s
60s
120s
300s
PARP2 KO RPE-1
+DMSO
+niraparib
DMSO
+niraparib
WT
XRCC1
R140A
B
PARP2 KO_hPARP2 + DMSO
PARP2 KO_hPARP2 + nira
PARP2 KO_hPARP2 RA + DMSO
PARP2 KO_hPARP2 RA + nira
Relative hPARP2 Intensity
Time (s)
C
Relative PARP2 intensity (max)
ns
ns
***
***
WT + DMSO
WT+ nira
R140A + DMSO
R140A + nira
D
Relative XRCC1 Intensity (Max)
***
ns
ns
***
WT + DMSO
WT+ nira
R140A + DMSO
R140A + nira
E
PARP1-/-
DKO
+PARP2RA
MMS
niraparib
75 KD
50 KD
75 KD
50 KD
15 KD
PARP2
XRCC1
α-Tubulin
H3
Soluble
Chromatin
F
PARP1/2 DKO + hCD8
PARP1/2 DKO + hCD8/hPARP2RA
Day 0
Day 6
87.7
90.0
90.7
91.0
SSC
hCD8
G
Relative Survival (%)
niraparib (μM)
WT
PARP1 KO
PARP1/2 DKO + hCD8
PARP1/2 DKO + hCD8/PARP2RA
(IC50=4.5)
(IC50=8.1)
(IC50=15.8)
(IC50=14.1)
***
***
ns
***
